# Immune cell dynamics in response to an acute laboratory stressor: a within-person between-group analysis of the biological impact of early life adversity

**DOI:** 10.1080/10253890.2022.2148100

**Published:** 2022-01

**Authors:** Laura Etzel, Abner T. Apsley, Brooke C. Mattern, Waylon J. Hastings, Thomas Heller, Nilam Ram, Sue Rutherford Siegel, Idan Shalev

**Affiliations:** a Department of Biobehavioral Health, The Pennsylvania State University, University Park, PA, USA; b Department of Psychology and Department of Communication, Stanford University, Stanford, CA, USA

**Keywords:** Early life adversity, acute stress, psychosocial stress, immune cell dynamics

## Abstract

Early life adversity (ELA) is a risk factor for early onset morbidities and mortality, a relationship that may be driven in part by immune system dysregulation. One mechanism of dysregulation that has yet to be fully examined in the context of ELA is alterations to immune cell dynamics in response to acute stress. Using a within-person between-group experimental design, we investigated stress-induced changes in immune cell populations, and how these changes may be altered in individuals with a history of ELA. Participants were young adults (*N* = 34, aged 18–25 years, 53% female, 47% with a history of ELA). Complete immune cell counts were measured at four time-points over a 5-hour window across two sessions (Trier Social Stress Test [TSST] vs. no-stress) separated by a week. Across all participants, total white blood cells increased over time (*F*(3,84)=38.97, *p* < .001) with a greater increase in response to the TSST compared to the no-stress condition at 240 minutes post-test (*b* = 0.43±.19; *t*(179)=2.22, *p* = .027). This pattern was mirrored by neutrophil counts. Lymphocyte counts were initially depressed by TSST exposure (*b* = −205±.67; *t*(184)= −3.07, *p* = .002) but recovered above baseline. ELA status was associated with higher stress-induced immune cell counts, a difference likely driven by increases in neutrophils (*F*(1,22)=4.45, *p* = .046). Overall, these results indicate differential immune cell dynamics in response to acute stress in individuals with a history of ELA. This points to altered immune system functioning in the context of stress, a finding that may be driving increased morbidity and mortality risk for ELA-exposed individuals.

## 1. Introduction

Exposure to early-life adversity (ELA; e.g. maltreatment, poverty) is a known risk factor for early-onset morbidities (Anda et al., 2010; Batten et al., 2004; Cohen et al., 2007; Eriksson et al., 2014). Links between ELA and future disease may be mediated by alterations in neuroendocrine (e.g. sympathetic nervous system [SNS] and hypothalamic-pituitary-adrenal [HPA] axis) responses to stress and immune system activity (Bunea et al., 2017; Elwenspoek et al., 2017; Hosseini-Kamkar et al., 2021; Nusslock & Miller, 2016). Disruptions to immune activity due to ELA have been documented across both innate and adaptive branches of the immune system. Chronic inflammation, altered responsivity to immune challenges, impaired latent viral control, and a disconnect between immune response and immune functionality (e.g. increased inflammation but decreased protection from pathogens) are all linked to early experiences of adversity (Cunningham et al., 2022; Elwenspoek et al., 2017; Baumeister et al., 2016; Tursich et al., 2014). Work remains to be done in mechanistically linking ELA to alterations of these large-scale immune processes.

One such pathway may be differential stress-induced proliferation and trafficking of immune cells. Immune cells are constantly trafficked in and out of the bloodstream, attached to and released from vascular walls, and newly produced via proliferation of hematopoietic stem cell (HSC) reserves (Ince et al., 2018). Stress hormones, including cortisol and catecholamines, released via HPA-axis and SNS activation respectively, influence proliferation and movement of immune cells, thus impacting total circulating supply (Ince et al., 2018; Heidt et al., 2014). Acute stress alterations of immune cell dynamics has been studied in healthy individuals. Medical students had higher circulating lymphocytes and granulocytes when they were on-duty versus off-duty days (Heidt et al., 2014). Healthy volunteers exposed to psychosocial stress experienced increased circulating lymphocytes 10 minutes post-stressor and subsequently falling below baseline by 120 minutes post-stress (Geiger et al., 2015). In these same healthy participants, exposure to the stressor induced an initial decrease in granulocytes 10 minutes post-stressor that recovered above baseline 120 minutes post-stress; no differences across the stressor were observed for monocytes (Geiger et al., 2015). In male first-time tandem parachutists, circulating lymphocytes and natural killer cells increased immediately prior to jumping subsequently dropping below baseline an hour post-jump (Schedlowski et al., 1993).

Work in animals provides a basis for exploring this phenomenon in ELA-exposed individuals. In mice, chronic variable stress increased circulating lymphocytes and neutrophils via SNS release of norepinephrine and stimulation of HSC activity (Heidt et al., 2014). A previous review indicated that in response to stress, immune cells are recruited to the bloodstream via demargination and proliferation of splenic hematopoietic reserves (Benschop et al., 1996). Recent animal work suggested catecholamine release due to repeated social stress enhances proliferation of immune cells from bone marrow and mobilizes progenitor cells to the spleen where they continue heightened immune cell production for weeks post-stress (McKim et al., 2018).

Data on stress and immune cell dynamics in ELA-exposed individuals is sparse. An examination of DNA methylation dynamics in adults with ELA reported differences in immune cell counts across psychosocial stress exposure including increased in total WBCs, lymphocytes and monocytes, and decreased granulocytes 10 minutes post-stress (Unternaehrer et al., 2012). However, this study had (1) no comparison group, (2) no within-person controls for confounds, such as diurnal immune cells rhythms, and (3) no significance tests of immune cell count differences.

We offer the following exploratory study as a call to action for researchers to examine stress-induced immune cell dynamics in existing datasets of ELA-exposed individuals. We tested differences in immune cell counts across exposure to a canonical laboratory stressor (compared with a resting control condition) between individuals with and without exposure to ELA (Shalev et al., 2020). The within-person control condition enables us to isolate the effects of acute stress while the between-person comparison provides for testing of theory about how ELA programs biological systems to operate differently to stress. We examined total white blood cell (WBC), lymphocyte, monocyte, and granulocyte change across time, and whether patterns of change differed in response to acute stress. We then characterized how stress-induced immune cell dynamics differ by ELA-status, hypothesizing that ELA-exposed individuals would evince differential immune cell counts when stressed compared to non-ELA exposed individuals. Given the paucity of data on stress-induced changes in immune cell counts across time within ELA-exposed individuals, we had no specific hypotheses on the direction of difference between ELA and control participants for each immune cell type.

## 2. Materials & methods

### 2.1. Participants

Participants were college students at the Pennsylvania State University, age 18–25 years, with no medical illness. Physical health was assessed by self-report and physical examination by trained nurses. Exposure to ELA was confirmed by a trained clinical interviewer conducting a phone interview using the Stressful Life Events Screening Questionnaire (SLESQ) (Goodman et al., 1998). Specifically, we asked respondents about their exposure to 11 specific and two general categories of events, such as the death of a parent or sibling, life-threatening accident, and sexual and physical abuse. Based on evidence that three or more traumatic events confers higher risk for disease (Felitti et al., 1998), and considering the severity of the traumatic events, participants who indicated exposure to at least three incidents up to age 18 years were classified as having ELA (see Shalev et al., 2020 for a full description of participant recruitment and methods) (Shalev et al., 2020). The SLESQ was also used to identify a control group of participants without a history of traumatic exposures, defined as having experienced 0 incidents up to age 18 years. The final sample included 34 participants, 16 of whom experienced early adversity (i.e. “ELA-group”) and 18 who did not (i.e. “control-group”) (mean age = 21.5, SD = 1.6; 53% female; 32% minority; see Table 1 for full sample demographics). The study was approved by the Ethics Committee at the Pennsylvania State University, registered at ClinicalTrials.gov (Identifier: NCT03637751), and all participants provided written informed consent. Participants received a modest monetary incentive for participation.

### 2.2. General produre

**Figure 1.**
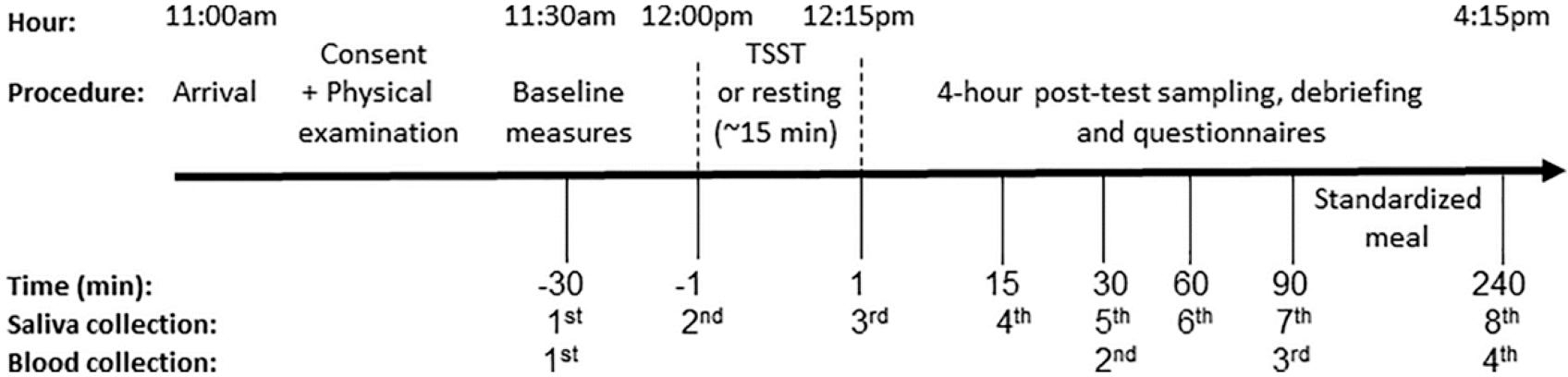
Study design for both sessions (TSST and no-stress condition). Sessions were separated by one week. Time is denoted as minutes measured from the 15-minute long TSST or resting condition (0 = midway point in session).

Participants made two visits to the Pennsylvania State’s Clinical Research Center between 11:00 am – 4:15 pm during weekdays, one week apart, on the same day of the week, with randomized counter-balanced ordering of the experimental manipulations (e.g. stress and no-stress conditions). Participants were given specific instructions to refrain from excessive physical activity on the day of the testing, consuming alcohol for 12 hours before their arrival, and eating and drinking (besides water) for 2 hours prior to the testing session. After arrival and consent, trained nurses completed a physical examination and inserted an IV catheter into the antecubital vein 30 minutes after arrival (30 minutes prior to testing). The experimental condition was always scheduled to begin at 12:00 pm to minimize the effects of circadian changes and was carried out as described previously (Shalev et al., 2020). In brief, when in the stress condition, participants completed the Trier Social Stress Test (TSST), a free speech and a mental arithmetic task of 10 minutes duration performed in front of a panel of two committee members (mixed gender) with a camera and microphone situated between the interviewers. When in the no-stress condition, participants sat in a room, read magazines, and refrained from stressful activities (e.g. cellphone use was restricted). Given the length of each testing session and the repeated collection of multiple blood samples, a standardized low-calorie meal was provided after the third blood draw at 1:45 pm (Figure 1 for outlined study design).

Additionally, for purposes related to the primary study objectives (Shalev et al., 2020 for details), saliva and blood pressure were collected across each session (Figure 1). Drawing from this data, we performed analyses of changes in cortisol and mean arterial pressure across session and time with the goal of validating the induction of stress across two major stress response systems, the HPA-axis and SAM (see Supplemental Digital Content A and B for full methods and results of these analyses).

### 2.3. Blood draws for immune cell counts

Blood draws for immune cells were taken at each session at: 30 minutes after arrival (30 minutes prior to testing), 30 (75 minutes after the first sample in the no-stress condition), 90, and 240 minutes post-test (Figure 1). A total of eight 4 ml EDTA tubes of whole blood were collected from each participant (4 tubes per session) and were sent to Quest Diagnostics on the same day for quantification of complete blood count (CBC) with differential using a standardized protocol. Analyses used absolute counts of total WBC, lymphocytes, monocytes, and granulocytes (i.e. neutrophils, eosinophils, and basophils).

### 2.4. Covariates

Age, socioeconomic status (SES; self-reported parent’s income level defined as upper, upper-middle, middle, lower-middle, or lower class), biological sex (male, female), minority status (self-identified non-Hispanic white vs. other), and body mass index (BMI) were obtained from baseline questionnaires and included as covariates as these variables are known to be associated with blood cell counts (Chen et al., 2006; Cheng et al., 2004; Choong et al., 1995; Desai et al., 2006; Pollitt et al., 2008; Starr & Deary, 2011).

### 2.5. Statistical analysis

Total WBC, lymphocytes, monocytes, neutrophils, and basophils were kept in original total count scale (total cells per μL). Total eosinophil counts were log-transformed to adjust for skewness. For ease of interpretation, binary predictor variables (e.g. ELA, sex, minority status, session order, session (TSST vs. no-stress) were centered on zero (e.g. dummy coded as −0.5 and 0.5), and continuous predictor variables (age, SES, BMI) were centered at sample grand means (age centered at 24.5 years, SES centered at 3 [“middle class”], BMI centered at 24.4). Although immune cell counts were missing for 10 of the 222 (4.5%) total observations (due to technical issues with either blood draws, shipping the blood, or analyses at Quest), full counts were available for 29 participants (13 ELA, 16 non-ELA; total number observations = 222; number of TSST session observations = 109). Baseline differences in immune counts across ELA status, sessions, and covariates were assessed using Wilcoxon rank-sum testing with Monte Carlo simulation of exact p-values at a sampling depth of 100,000; with significance tests all robust across sample depths of 10,000 to 1,000,000.

Immune cell counts were examined separately for total WBCs, lymphocytes, monocytes, neutrophils, basophils, and eosinophils using repeated measures ANOVA, with time invoked as a categorical variable where the baseline assessment occurring 30 minutes prior to the experimental manipulation was used as the reference category. These repeated measures ANOVAs were conducted within a multilevel modeling framework that accommodates both the unevenly spaced time intervals and the complex covariance structure of the data (Hox et al., 2018; Gueorguieva & Krystal, 2004). Models included: a model that used time and session variables to examine how change in each outcome differed across experimental conditions, a model conditioned on ELA, and a fully adjusted model that additionally conditioned on covariates (see Supplemental Digital Content for complete model specifications). All analyses were conducted in SAS v9.4 (SAS Institute Inc., Cary, NC) using PROC MIXED for the multilevel models with maximum likelihood estimation and incomplete data (<5%) accommodated under missing at random assumptions. Statistical significance was evaluated at *α* = 0.05. To ensure that we were able to identify all potential associations worthy of future study, no adjustments were made for multiple comparisons in this exploratory study.

## 3. Results

### 3.1. Sample demographics and descriptive statistics

Demographics for the full sample and separately for the ELA and control groups are shown in Table 1. As previously reported, the ELA and control groups did not differ with respect to age, SES, % minority, % female (Shalev et al., 2020). However, there was a significant difference in BMI, with the ELA group having a lower average BMI (22.4) compared to the control group (26.3) (WMW estimated *p*-value <.001). Across all 8 time-points in both sessions, total WBC counts were marginally correlated with biological sex (*r* = −0.35, *p* = .06), suggesting higher levels of WBCs in females compared to males. This effect may be driven by differential granulocyte activity in females compared to males (Supplemental Digital Content Table S3), which is in line with previous literature on sex differences in immune response to challenge (Klein & Flanagan, 2016). Total eosinophil counts were correlated with SES (*r* = −0.33, *p* = .013), indicating higher eosinophil counts for those with lower reported SES, while higher basophil counts were significantly correlated with older age (*r* = 0.41, *p* = .001), ELA-status (*r* = 0.50, *p* < .001), female sex (*r* = −0.49, *p* < .001), and lower BMI (*r* = −0.32, *p* = .015) (Supplemental Digital Content Table S3 for complete correlations between all study variables, and Table S4 for session by time summary statistics for immune cell counts).

### 3.2. Validation of the acute stress induction procedure

Induction of acute stress by the TSST was validated through assessments of HPA-axis and SNS activation, specifically, salivary cortisol and mean arterial pressure (MAP). On average, there was a significant within-person cortisol response to the TSST compared to the no-stress condition (Session, *F* = 28.05, *p* < .001; see Table S1). Exposure to the TSST was significantly associated with higher peak cortisol (*b* = 0.51±.10; *t*(436) =5.30, *p* < .001) and a steeper decline in cortisol over time in the TSST session compared to the no-stress session (*b* = −0.006±.001; *t*(436) = −5.60, *p* < .001). Inclusion of random effects of session and random slopes for reactivity and recovery significantly improved model fit, indicating a significant amount of variability in the effect of session and time on cortisol reactivity between individuals. There were no differences in cortisol response by ELA-status.

MAP responses also differed between the TSST and no-stress conditions (Session, *F* = 21.54, *p* < .001; see Table S2), exhibited a significant change across time (Time, *F* = 15.51, *p* < .001), and differences in change across time between the TSST and no-stress conditions (Session × Time, *F* = 26.07, *p* < .001). Exposure to the TSST was significantly associated with higher MAP at one minute prior to TSST exposure (*b* = 7.46 ± 2.22 *t*(93)=3.36, *p* = .001) and one minute post-TSST (*b* = 7.94 ± 2.22; *t*(93)=3.58, *p* < .001). Notably, the session by time interaction differed with ELA (Session × Time × ELA, *F* = 3.21, *p* = .026). Compared to the control group, the ELA group exhibited greater increases in MAP in response to the TSST at one minute prior (*b* = 7.99 ± 3.26; *t*(93)=2.45, *p* = .016) and one minute post-TSST (*b* = 8.88 ± 3.26; *t*(93)=2.72, *p* = .007), as well as a continued elevation of MAP post-TSST at 15 minutes (*b* = 7.79 ± 3.26; *t*(93)=2.39, *p* = .019). For full cortisol and MAP analyses, see Supplemental Digital Content Tables S1 and S2, respectively.

### 3.3. Analysis of baseline immune cell counts

Baseline total WBC counts of the ELA and control groups did not differ at the no-stress (*t* = 0.92; *p* = .36) or TSST session (*t* = 1.33; *p* = .19). There were also no differences between the two groups in baseline lymphocytes (no-stress: *t* = 0.34; *p* = .73; TSST: *t* = 0.04; *p* = .96), monocytes (no-stress: *t* = 0.58; *p* = .56; TSST: *t* = 1.28; *p* = .21), neutrophils (no-stress: *t* = 1.46; *p* = .14; TSST: *t* = 0.13; *p* = .90), or eosinophils (no-stress: *t* = 0.20; *p* = .83; TSST: *t* = 0.54; *p* = .59). However, there were group differences in baseline basophil at both sessions, with the ELA-group having 63–88% higher basal basophil levels than the control group (no-stress: *t* = 3.30; *p* = .002; TSST: *t* = 2.58; *p* = .017).

### 3.4. Effect of acute stress and ELA status on immune cell counts

#### 3.4.1. Total white blood cell count

**Figure 2.**
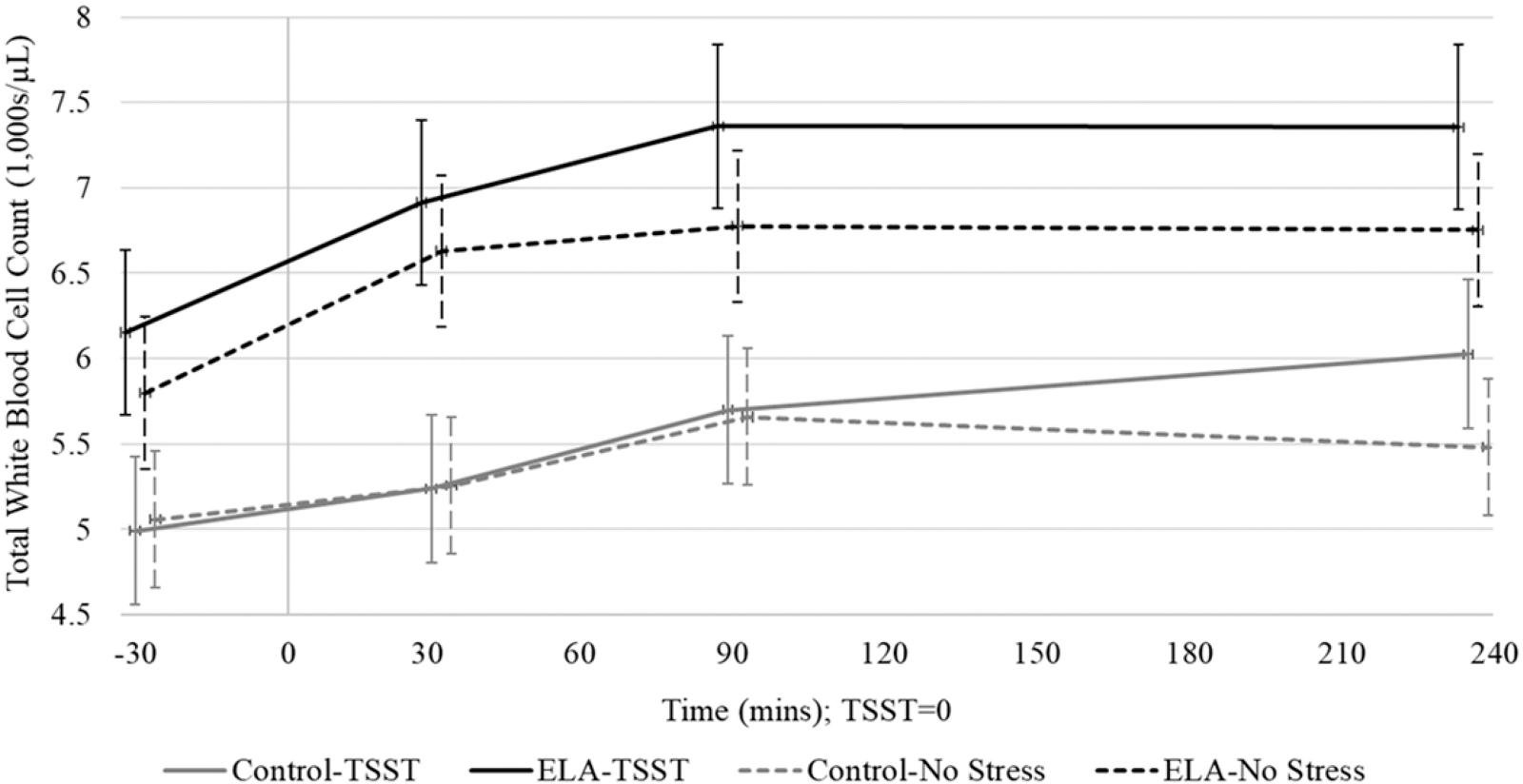
Total white blood cell counts (Est ± SE) across time within the ELA group vs. control group in the TSST and no-stress sessions. Time is denoted as minutes measured from the 15-minute long TSST or resting condition (0 = midway point of session).

With respect to the impact of acute stress, there was no significant within-subject main effect of TSST versus no-stress session on total WBC counts, though there was a significant increase over time from baseline across both sessions (*F*(3,84)=38.97, *p* < .001). On average, total WBCs increased by 9, 16, and 17% at 30, 90, and 240 minutes, respectively (Figure 2). There was a trend toward differences in Session × Time interaction (*F*(1,179)=2.25, *p* = .084), reaching significance at 240 minutes (*b* = 0.43±.19; *t*(179)=2.22, *p* = .027), indicating on average participants had higher total WBCs at 240 minutes in the TSST session compared to the no-stress session (see Table 2 for Final Models). With respect to the impact of ELA status, there was a trend toward a significant main effect of ELA on total WBC counts (*F*(1,22)=3.58, *p* = .071). The interaction between ELA × Time was significant at 30 minutes (*b* = 0.57±.19); *t*(179) =3.03, *p* = .002) and 90 minutes (*b* = 0.44±.19); *t*(179)=2.32, *p* = .021), indicating higher total WBCs in the ELA group across both sessions at 30 – 90 minutes compared to baseline.

#### 3.4.2. Lymphocytes

**Figure 3.**
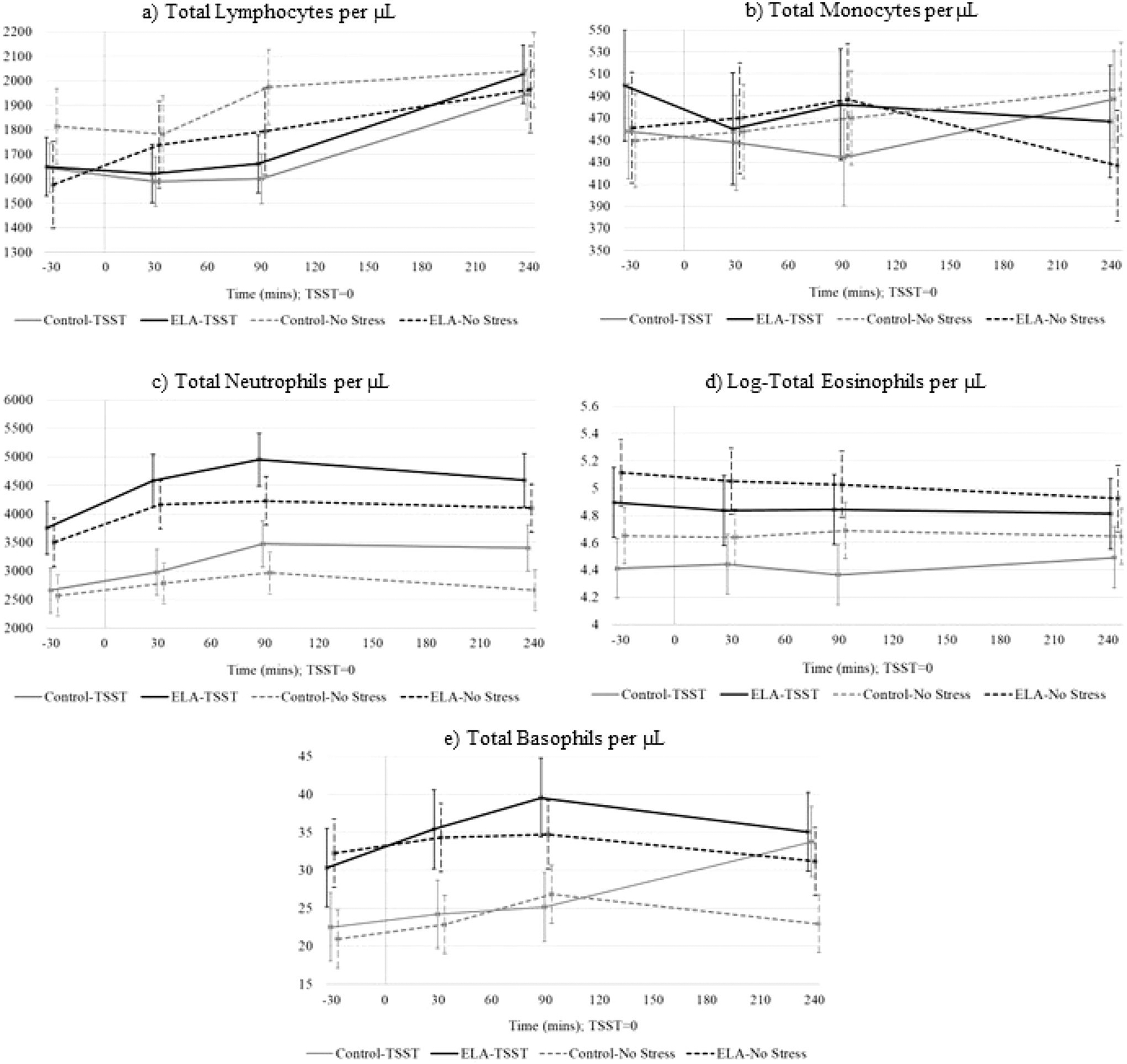
Immune cell counts (Est ± SE) across time for ELA-group vs. control-group within the TSST and no-stress conditions. Time is denoted as minutes measured from the 15-minute long TSST or resting condition (0 = midway point of session).

With respect to the impact of acute stress, there was no significant within-subject main effect of TSST versus no-stress session on total lymphocyte counts (*F*(1,184)=1.82, *p* = .17), however, lymphocytes significantly changed across time (*F*(3,84)=39.99, *p* < .001). Specifically, there was a significant interaction between Session × Time (*F*(3,177)=5.30, *p* = .001) such that in the no-stress session there was an average increase in lymphocytes of 16.7%, whereas in the TSST session there was a 7% drop in lymphocytes at 90 minutes post-TSST (*b* = −205 ± 0.67; *t*(184) = −3.07, *p* = .002), followed by a recovery overshooting baseline levels by 16% at 240 minutes post-TSST (Figure 3(a)). There was no main effect of ELA-status on lymphocytes (*F*(1,2)=0.04, *p* = .83) across either session and no significant interactions.

#### 3.4.3. Monocytes

With respect to the impact of acute stress, monocyte counts did not differ significantly within-subject across the TSST and no-stress sessions (*F*(1,177)=0.02, *p* = .89) or over time (*F*(3,84)=0.26, *p* = .85) (Figure 3(b)). As well, the non-significant Session × Time interaction (*F*(3,177)=1.24, *p* = .29) indicated that monocytes were not influenced by the stress induction. With respect to the impact of ELA status, there was no main effect of ELA (*F*(1,22)=0.01, *p* = .92), though the interaction between ELA × Time was significant (*F*(3,177)=3.48, *p* = .017). On average, across both sessions, ELA-exposed individuals had lower monocyte counts than controls at 240 minutes (*b* = −71 ± 0.26; *t*(177) = −2.69, *p* = .007).

#### 3.4.4. Neutrophils

With respect to the impact of acute stress, within-subject effect showed higher neutrophil counts in the TSST session compared to the no-stress session (*F*(1,181)=4.98, *p* = .026). There was also a significant effect of time (*F*(3,84)=25.75, *p* < .001) with neutrophils increasing by 16, 25, and 18% above baseline at 30, 90, and 240 minutes respectively. Interactions between Session × Time at 90 minutes (*b* = 438±.185; *t*(181)=2.37, *p* = .018) and 240 minutes (*b* = 446 ± 0.188; *t*(181)=2.37, *p* = .018) indicate greater increases in neutrophils in response to the TSST compared to the no-stress session. With respect to the impact of ELA status, there was a significant effect of ELA on total neutrophils (*F*(1,22)=4.45, *p* = .046), with ELA-exposed individuals averaging 17% higher neutrophil counts (Figure 3(c)). Interaction terms for ELA × Time showed a significant effect at 30 minutes post-TSST (ELA × Time = 30 minutes, *b* = 474 ± 184; *t*(181)=2.58, *p* = .010).

#### 3.4.5. Eosinophils

With respect to the impact of acute stress, there was a significant within-subject main effect on eosinophil counts (*F*(1,187)=6.14, *p* = .010) such that exposure to the TSST was associated with a 14% lower eosinophil counts (*b* = −0.21 ± 0.08; *t*(187)= −2.48, *p* = .014) (Figure 3(d)). Eosinophils did not change significantly over time (*F*(3, 84) =0.26, *p* = .85), and all interaction terms for Session × Time were non-significant. With respect to the impact of ELA, there were no significant differences in eosinophil counts (*b* = 0.39 ± 0.35; *t*(22)=1.12, *p* = .27) and all interaction terms for ELA × Session or Time were not significant.

#### 3.4.6. Basophils

With respect to the impact of acute stress, there was a within-subject trend toward higher basophil counts in the TSST session (*F*(1,184)=3.02, *p* = .084) and a significant effect of Time (*F*(3,84)=3.60, *p* = .009). At 90 minutes, there was a significant increase of 17% above baseline across both groups (*b* = 5.0 ± 1.5; *t*(84)=3.26, *p* = .001). By 240 minutes, basophils were still elevated 16% above baseline (*b* = 4.23 ± 1.6; *t*(84)=2.69, *p* = .008). Interactions for Session × Time reached significance at 240 minutes (*b* = 7.33 ± 3.1; *t*(84)=2.33, *p* = .020). This effect may in part be driven by the sharp increase in basophil counts within controls in the TSST session (Figure 3(e)). With respect to the impact of ELA status, there were no significant differences in basophil counts (*F*(1,22)=2.67, *p* = .11). Interaction terms for ELA × Session or Time were not significant.

## 4. Discussion

This study used a within-person between-group repeated measures design to investigate immune cell dynamics, captured as immune cell counts, in response to acute stress in individuals exposed to ELA and a control group. Overall, we found a pattern of increased total WBCs across time and session, likely driven by increases in neutrophils. The increase in neutrophils seemed to be enhanced for individuals with ELA, such that they experienced greater increases in neutrophils than controls. Our finding of increased total WBC counts across both the acute stress and no-stress conditions may be due in part to either the initial stress of the venipuncture, or may reflect typical diurnal changes in WBCs (Born et al., 1997). At 240 minutes post-TSST, we observed a significant increase in total WBCs due to the TSST across both ELA and control groups which is consistent with literature finding an increase in mobilization of immune cells overall due to acute stress in both animal models and humans (Schedlowski et al., 1993; Dhabhar et al., 2012).

The pattern of change observed in total WBC counts was most clearly replicated in neutrophil counts, which also exhibited a significant increase over time during both the no-stress and TSST sessions. Neutrophils are the most abundant type of WBC (Nathan, 2006), and thus may be responsible for a large share of the increase in WBCs across both sessions seen here. Even so, there were higher neutrophil counts and a more robust increase in neutrophils above baseline during the TSST session compared to the no-stress session, indicating an effect of acute stress on neutrophil trafficking similar to those observed in animal models (Dhabhar et al., 1994; Dhabhar et al., 1995). Basophil counts increased across time in both sessions, with a trend toward greater increases in the TSST session. Eosinophil counts were lower across both ELA and control groups in the TSST session, though we did not see an effect of time across either session.

In the no-stress condition, lymphocytes followed a similar pattern of increase over time mirroring that of total WBCs. However, in the TSST session across both groups, lymphocyte trafficking was initially depressed before recovering above baseline levels. Existing literature points to increases in lymphocytes post-stress exposure. Our finding of an initial depression in lymphocytes prior to an increase may be due to the type of stressor (e.g. TSST vs. exercise and emotional stressors) or the timing of the stressor and immune cell counts measurement. Monocytes did not demonstrate a clear pattern in our study, perhaps due to the within-person large variation in monocyte trafficking over time across both sessions.

Differences in immune cell dynamics as a function of ELA status were observed for a few cell types. The ELA group exhibited higher increases in total WBCs 30–90 minutes compared to the control group, irrespective of session. Examination of Figure 3 demonstrates that these differences in WBCs appear to be driven by differences in counts of neutrophils, basophils, and eosinophils, which all tended to be higher in the ELA group, although only differences in neutrophils were statistically significant. Differences in the trafficking of WBCs and immune subsets in response to stress can have health implications for individuals exposed to ELA. Neutrophils are the major pathogen fighting immune cells, functioning as regulators of innate and adaptive immune responses (Mayadas et al., 2014). Neutrophils are major contributors in the pathogenesis of various autoimmune diseases, autoinflammatory syndromes, and cardiovascular diseases (Dahdah et al., 2022; Kaplan, 2013; Wouters et al., 2021). Eosinophils are also involved in the immunological regulation of both innate and adaptive immunity (Wen & Rothenberg, 2016). Eosinophils are responsible for tissue damage and inflammation in many diseases and are involved in the development of type 2 diabetes and atherosclerosis (metabolic homeostasis) (Jacobsen et al., 2012), chronic diseases which individuals exposed to ELA are at increased susceptibility to develop (Rich-Edwards et al., 2010; Su et al., 2015). Basophils are involved in inflammatory responses, as well as the development of allergic diseases. Taken together, this may be indicative of chronic hyperactivity of the distinct elements of the immune system at rest and in response to acute stress in individuals with a history of ELA, as has been previously suggested (Danese et al., 2007).

Strengths of this study included the use of a within-person, between-group laboratory-based experimental design that allowed for comprehensive study of differences in stress-induced immune cell dynamics. The collection of multiple repeated measurements across time during multiple sessions accommodated between-person differences in underlying biology and increased statistical power for detecting potential associations worthy of further study. The hypotheses investigated here were secondary investigations using an existing dataset that had >90% power to detect small associations (0.08 ≤ *r* ≥ 0.14) in primary outcomes (see Shalev et al., 2020 for more information). Preliminary analyses on a subset of this cohort (*N* = 12) found evidence of higher cortisol responses and lower glucocorticoid receptor gene expression for individuals with ELA in response to the TSST (Shalev et al., 2020). Though we observed a significant cortisol response to the TSST, we did not replicate the previous differential cortisol response by ELA-status findings in the larger cohort. We did see evidence of differential blood pressure responses for individuals with ELA possibly pointing to differences in stress induced SNS responses.

We acknowledge limitations for the present study. First, although we used a robust design, the study sample is still relatively small and homogenous. Although comparable in size and findings to previous studies on the topic (Sgoutas-Emch et al., 1994; Herbert et al., 1994), our results should be interpreted cautiously and, primarily, as a call to action for analyses with larger and more diverse samples. For instance, despite documented correlations between increased BMI and exposure to adversity, within our sample we found that the ELA group had a significantly lower BMI than the control group. Given that higher BMI is associated with higher WBC counts (Kabat et al., 2017; Dixon & O’Brien, 2006), this does not diminish our findings, rather it underscores the need for a larger and more diverse sample in future studies. Second, due to the small sample size, we were limited in the analyses we could perform. For instance, there are well documented sex differences in immune function between males and females (Klein & Flanagan, 2016), but we did not have sufficient sample size to examine sex differences in immune cell dynamics. Instead, we controlled for sex in all analyses. Future work in larger samples might use similar multimodal designs (saliva and blood) to also investigate time-lagged immune cell dynamics as a function of stress-induced HPA-axis responses as this is a key pathway likely to be altered in individuals with ELA exposure (Hosseini-Kamkar et al., 2021). Third, we focused on the main subsets of immune cell types that are routinely assessed via a CBC panel, and were thus unable to parse the effects of acute stress and ELA on subclasses of lymphocytes and monocytes that vary in their responses to acute stress (Herbert & Cohen, 1993). Fourth, ELA was characterized as a dichotomous variable thus collapsing potentially important differences in severity, type, and number of adversities within our sample. We are underpowered to examine these factors and future work should prioritize recruitment of a sample that can parse these critical elements of adversity exposure.

## 5. Conclusion

We employed a unique repeated measures design to investigate the impact of an acute stress paradigm on immune cell dynamics. Our findings replicate elements of previous work demonstrating altered immune cell trafficking in response to acute stress. Our results highlight specific variations in immune cell trafficking in individuals with a history of ELA, which may point to differential immune system functioning in the context of acute stress. This differential functioning may in turn be driving some of the increases in morbidity and mortality observed in ELA-exposed individuals. We believe our findings should be viewed as a call to action for researchers to replicate tests in existing datasets of stress induction for individuals with ELA with larger and more diverse samples.

## Supplementary Material

Supplemental Digital Content

## Figures and Tables

**Table 1.** Summary statistics for the sample. Immune cell counts are averaged across all 8 time-points in both sessions.

Variable, mean (SD)	Total (*N* = 34)	ELA (*N* = 16)	Control (*N* = 18)	*p*-Value diff

Age	21.5 (1.6)	21.8 (1.8)	21.2 (1.3)	.63
SES (average)	3.26 (0.8)	3.19 (1)	3.33 (.6)	.71
% Minority Status	32%	19%	44%	.15
Sex Male (Female)	16 (18)	7 (9)	9 (9)	.74
Body Mass Index	24.4 (3.8)	22.4(3.3)	26.3 (3.4)	<.001^a^
WBCs (10^3^ cells per μL)	5.99 (1.34)	6.49 (1.58)	5.58 (0.99)	.12
Lymphocytes (cells per μL)	1762 (334)	1866 (358)	1676 (297)	.068
Monocytes (cells per μL)	474 (123)	491 (143)	460 (107)	.53
Neutrophils (cells per μL)	3591 (1207)	3950 (1357)	3300 (1022)	.19
Eosinophils (cells per μL)	138 (106)	136 (76)	140 (129)	.55
Basophils (cells per μL)	30 (17)	39 (17)	23 (13)	.006

a Bold values indicate significance as follows:

† *p*<.10

* *p*<.05

** *p*<.01

*** *p*<.001.

**Table 2.** Final best fit models for each cell type.

Final models	Total WBC Estimate (SE)	Lymphocytes Estimate (SE)	Monocytes Estimate (SE)	Neutrophils Estimate (SE)	Log-eosinophils Estimate (SE)	Basophils Estimate (SE)

Fixed effects						
Intercept	5.41*** (0.28)	1669*** (84)	475*** (24)	3103*** (251)	4.97*** (0.14)	28.6*** (2.8)
Session	0.14 (.22)	−57 (101)	23 (23)	171 (221)	−0.21* (0.08)	− 0.1 (2.3)
Time (30 mins)	0.51*** (0.09)	8(33)	−8 (13)	506*** (92)	−0.02 (0.06)	2.7† (1.5)
Time (90 mins)	0.87*** (0.09)	85* (33)	1 (13)	785*** (93)	−0.03 (0.06)	5.0** (1.5)
Time (240 mins)	0.90*** (0.10)	321*** (34)	2 (13)	566*** (94)	−0.05 (0.06)	4.3** (1.5)
Interaction: session × time (30 mins)	− 0.01(0.19)	−102 (65)	−33 (26)	128 (183)	−	1.3(3.1)
Interaction: session × time (90 mins)	0.17 (0.19)	−205** (67)	−43† (26)	438* (185)	−	1.5(3.1)
Interaction: session × time (240 mins)	0.43† (0.19)	35 (68)	−7 (27)	446* (188)	−	7.3* (3.1)
ELA-status	0.95 (0.69)	35 (165)	27 (71)	998 (619)	0.39(.35)	9.9(6.0)
Interaction: session × ELA	0.42 (0.44)	-	30 (45)	-	-	-
Interaction: ELA × time (30 mins)	0.57** (0.19)	-	−14 (26)	474* (184)	-	-
Interaction: ELA × Time (90 mins)	0.44* (0.19)	-	6(26)	358† (185)	-	-
Interaction: ELA × Time (240 mins)	0.35† (0.19)	-	−71** (26)	312† (188)	-	-
Interaction: session × ELA × time (30 mins)	−0.12 (0.38)	-	−29 (52)	-	-	-
Interaction: session × ELA × time (90 mins)	0.12 (0.38)	-	1 (52)	-	-	-
Interaction: session × ELA × time (240 mins)	−0.36 (0.39)	-	19 (53)	-	-	-
Age	−0.02 (0.19)	57 (50)	13 (20)	−139 (179)	0.28* (0.10)	2.8 (1.8)
Sex	−0.97 (0.51)	−106 (129)	−27 (53)	−980* (462)	0.75** (0.26)	−10.6* (4.6)
Minority	0.20 (0.49)	10 (127)	−24 (51)	337 (441)	0.43(.25)	1.1 (4.4)
SES	0.02 (0.31)	44 (81)	−23 (32)	22 (283)	−0.41* (0.16)	−4.6 (2.8)
BMI	0.09 (0.09)	−33 (23)	−3 (10)	127 (84)	0.02 (0.05)	−0.9 (0.84)
Random effects						
Subject-level variance	1.29 (0.35)	122851 (35045)	12873 (3553)	1046217 (286159)	0.34 (0.09)	129 (37)
Session-intercept Covariance	0.22 (0.23)	−111358 (42040)	224 (2155)	212794 (208364)	0.04 (0.05)	44 (19)
Session variance	0.83 (0.26)	229497 (69686)	4964 (2162)	884145 (272489)	0.15 (0.05)	20 (15)
Residual variance	0.25 (0.03)	30762 (3454)	4678 (525)	235672 (26115)	0.08 (0.009)	66 (7)

See supplemental digital content Tables S5–S10 for model iterations for each cell type. Bold values indicate significance as follows:

† *p* < .10

* *p* < .05

** *p* < .01

*** *p* < .001.
