# Supplementary material for: Immune cell dynamics in response to an acute laboratory stressor: a within-person between-group analysis of the biological impact of early life adversity
Source: Stress. Author manuscript; Available in PMC 2023 Jan 1. (PMC9704543; doi:10.1080/10253890.2022.2148100)
Supplement: Supplemental Digital Content [file NIHMS1852122-supplement-Supplemental_Digital_Content.docx]

**Supplemental Digital Content**

A. Cortisol Analysis

*Cortisol Collection and Statistical Analysis*

Salivette swabs (Sarstedt, Germany) were used to collect saliva for salivary cortisol across the following 7 time-points: 30 minutes after arrival, 1 minute prior to the experimental condition, immediately after, and 15, 30, 60, and 90 minutes post-experimental condition. Saliva samples were kept at room temperature throughout the session, were immediately centrifuged at the end of the session at 3000 rpm at 24°C for 15 minutes, and then stored at -80°C until assayed. Salivary cortisol was assayed in duplicate through an enzyme immunoassay protocol (Salimetrics) with known controls. The lower detection limit of the assay is <0.007 ug/dL. Intra-assay CV was 10.38% across all samples and inter-assay CV 10.47% across 10 plates.

Prior to analysis, cortisol concentrations were log-transformed to adjust for skewness. Binary predictor variables (e.g. ELA, sex, minority status, session (TSST vs. no-stress) were centered on zero (e.g. dummy coded as -0.5 and 0.5), and continuous predictor variables (age, SES, BMI) were centered at sample grand means (age centered at 24.5 years, SES centered at 3, BMI centered at 24.4). Cortisol data was available for 34 participants (16 ELA, 18 non-ELA; total number of observations = 485; number of TSST session observations = 238). Tests for outliers identified one cortisol value greater than 3SD of mean values (observation was dropped from analysis).

Changes in cortisol were examined using a landmark registration approach. First, each individual’s peak stress-induced cortisol was identified as the first post-stress sample followed by either decline or plateau of less than 10% change (see full discussion of landmark registration with cortisol data in [^[[1]](#footnote-1)^]). Then, two time variables were created that separated and captured individuals’ reactivity to the stressor and recovery from the stressor. Specifically a reactivity slope was operationalized using a time-to-peak variable that indicated the number of minutes until the peak: increasing negative values before the peak and zero after the peak, and a recovery slope was operationalized using a time-since-peak variable that indicated the number of minutes since the peak: zero before the peak and increasing positive values after the peak.

*Results:*

On average for the sample, there was a significant within-person response to the TSST compared to the no-stress condition (Session, F=28.05, p<.0001; see Table S1). Exposure to the TSST was significantly associated with higher peak cortisol (b=0.51±.10; *t*(436)=5.30, p<.0001). Variability in cortisol reactivity and recovery slopes was tested using multiphase growth models. In all models, there was a significant change in cortisol post-experimental manipulation (e.g. recovery). Both the Recovery slope and the interaction term for Session X Recovery were significant (b=-0.009±.0007; *t*(436)=-11.74, p<.0001, and b=-0.006±.001; *t*(436)=-5.60, p<.0001, respectively), meaning there was a significant change in cortisol recovery across both sessions, with the overall decline in cortisol observed during recovery being steeper in the TSST session than in the no-stress session. Averaged across both sessions, there was no significant change in cortisol pre-experimental manipulation (Reactivity, b=0.0005±.002; *t*(436)=0.26, p=0.80); however, there was a significant difference in change in cortisol during the reactivity phase between sessions (Session X Reactivity b=0.01±.002; *t*(436)=6.15, p<.0001), indicating an increase in cortisol pre-stress exposure and a decrease in cortisol pre-no-stress exposure. Inclusion of random effects of session and random slopes for reactivity, and recovery significantly improved model fit (LRT: -2LL reduced model = 565, -2LL full model =517.1 (difference = 47.9, df=5) p<.0001), indicating a significant amount of variability in the effect of session and time on cortisol reactivity between individuals. There was significant covariation between peak cortisol (intercept) and the reactivity slope (b=0.004±.002; *z*=2.59 p=.009) indicating higher peak cortisol was associated with steeper activation slopes.

There was no significant main effect of ELA on cortisol peak (intercept), reactivity, or recovery slopes. Tests for sex differences on cortisol similarly yielded no significant findings. Tests for differences by the interaction of ELA X Sex did not meet significance cut-offs but pointed to a possible difference in recovery slope across sessions, with males having a steeper slope than females (b=-0.004±.002; *t*=-1.74 p=.08).

The final simplified composite cortisol model was of the form:

| $Log\text{-}{Cortisol}_{ij}=\beta_{0i}+\beta_{1}\left( {TimeToPeak}_{ij} \right)+\beta_{2}\left( {TimeSincePeak}_{ij} \right)+\beta_{3}\left( {Session}_{j} \right){+\beta_{4}\left( {TimeToPeak}_{ij} \right)\left( {Session}_{j} \right)+\beta_{5}\left( {TimeSincePeak}_{ij} \right)\left( {Session}_{j} \right)+\beta_{8}\left( {Age}_{j} \right)+\beta_{9}\left( {Sex}_{j} \right)+\beta_{10}\left( {Minority}_{j} \right)+\beta_{11}\left( {SES}_{j} \right)+\beta_{12}\left( {BMI}_{j} \right)+\mu_{0j}{+ \mu}_{1j}\left( {TimeToPeak}_{ij} \right)+\mu_{2j}\left( {TimeSincePeak}_{ij} \right)+\mu_{3j}\left( {Session}_{j} \right)+ e}_{ij}$ | (1) |
| --- | --- |

Table S1. Log-transformed cortisol predicted by session and reactivity and recovery time slopes (†p<.10, *p<.05, **p<.01, ***p<.001).

| **Log-transformed Cortisol** | Model 1 | Model 2 | Model 3 | Model 4 | Model 5 | Model 6 | Model 7 | Final Model |
| --- | --- | --- | --- | --- | --- | --- | --- | --- |
|  | Estimate (SE) | Estimate (SE) | Estimate (SE) | Estimate (SE) | Estimate (SE) | Estimate (SE) | Estimate (SE) | Estimate (SE) |
| Fixed Effects |  |  |  |  |  |  |  |  |
| Intercept (Peak) | **-1.99***(.08)** | **-1.79***(.09)** | **-1.79***(.09)** | **-1.79***(.09)** | **-1.79***(.09)** | **-1.79***(.09)** | **-1.85***(.10)** | **-1.85***(.10)** |
| Session | -- | **0.27***(.04)** | **0.27***(.04)** | **0.49***(.06)** | **0.49***(.06)** | **0.51***(.10)** | **0.51***(.10)** | **0.51***(.10)** |
| Time to Peak (Reactivity) | -- | 0.0008(.002) | 0.0008(.002) | 0.0007(.001) | 0.0007(.001) | 0.0004(.002) | 0.0005(.002) | 0.0005(.002) |
| Time since Peak (Recovery) | -- | **-0.008***(.0007)** | **-0.008***(.0007)** | **-0.008***(.0007)** | **-0.008***(.0007)** | **-0.009***(.0007)** | **-0.009***(.0007)** | **-0.009***(.0007)** |
| ELA-status | -- | -- | -0.16(.16) | -0.16(.16) | -0.16(.17) | -0.15(.17) | -0.15(.22) | -0.16(.18) |
| Interaction: Session x Reactivity | -- | -- | -- | **0.01**(.003)** | **0.01**(.003)** | **0.01***(.002)** | **0.01***(.002)** | **0.01***(.002)** |
| Interaction: Session x Recovery | -- | -- | -- | **-0.006***(.001)** | **-0.006***(.001)** | **-0.006***(.001)** | **-0.006***(.001)** | **-0.006***(.001)** |
| Interaction: Status x Session | -- | -- | -- | -- | -0.10(.08) | -0.08(.18) | -0.08(.18) | -- |
| Interaction: Status x Reactivity | -- | -- | -- | -- | 0.001(.002) | 0.001(.004) | 0.001(.004) | -- |
| Interaction: Status x Recovery | -- | -- | -- | -- | 0.00001(.001) | -0.0004(.001) | -0.0004(.001) | -- |
| Age | -- | -- | -- | -- | -- | -- | 0.04(.05) | 0.04(.05) |
| Sex | -- | -- | -- | -- | -- | -- | 0.17(.15) | 0.17(.15) |
| Minority | -- | -- | -- | -- | -- | -- | 0.05(.16) | 0.05(.16) |
| SES | -- | -- | -- | -- | -- | -- | **0.26(.10)†** | **0.26†(.10)** |
| BMI | -- | -- | -- | -- | -- | -- | -0.01(.02) | -0.01(.02) |
| Random Effects |  |  |  |  |  |  |  |  |
| Subject-level Variance | 0.20(.05) | 0.21(.06) | 0.21(.06) | 0.20(.05) | 0.20(.05) | 0.26(.07) | 0.30(.09) | 0.30(.09) |
| Session-Intercept Covariance | -- | -- | -- | -- | -- | 0.06(.05) | 0.10(.06) | 0.10(.06) |
| Session | -- | -- | -- | -- | -- | 0.25(.07) | 0.25(.07) | 0.25(.07) |
| Reactivity-Intercept Covariance | -- | -- | -- | -- | -- | 0.003(.001) | 0.004(.002) | 0.004(.001) |
| Session-Reactivity Covariance | -- | -- | -- | -- | -- | 0.001(.001) | 0.001(.001) | 0.001(.001) |
| Time to Peak (Reactivity) | -- | -- | -- | -- | -- | 0.0001(.00004) | 0.0001(.00004) | 0.0001(.00004) |
| Recovery-Intercept Covariance | -- | -- | -- | -- | -- | -0.0001(.0004) | -0.0004(.0004) | -0.0004(.0004) |
| Session-Recovery Covariance | -- | -- | -- | -- | -- | -0.0005(.0004) | -0.0005(.0004) | -0.0005(.0004) |
| Reactivity-Recovery Covariance | -- | -- | -- | -- | -- | -0.00001(.00001) | -0.00001(.00001) | -0.00001(.00001) |
| Time since Peak (Recovery) | -- | -- | -- | -- | -- | 0.00001(.000004) | 0.00001(.000004) | 0.00001(.000004) |
| Residual Variance | 0.27(.02) | 0.19(.01) | 0.19(.01) | 0.18(.01) | 0.18(.01) | 0.09(.007) | 0.09(.007) | 0.09(.007) |
| AIC/BIC | 819/822 | 661/670 | 662/678 | 646/659 | 650/668 | 490/522 | 495/534 | 489/524 |
| LRT p-value | -- | -- | -- | -- | -- | p<.0001 | -- | -- |


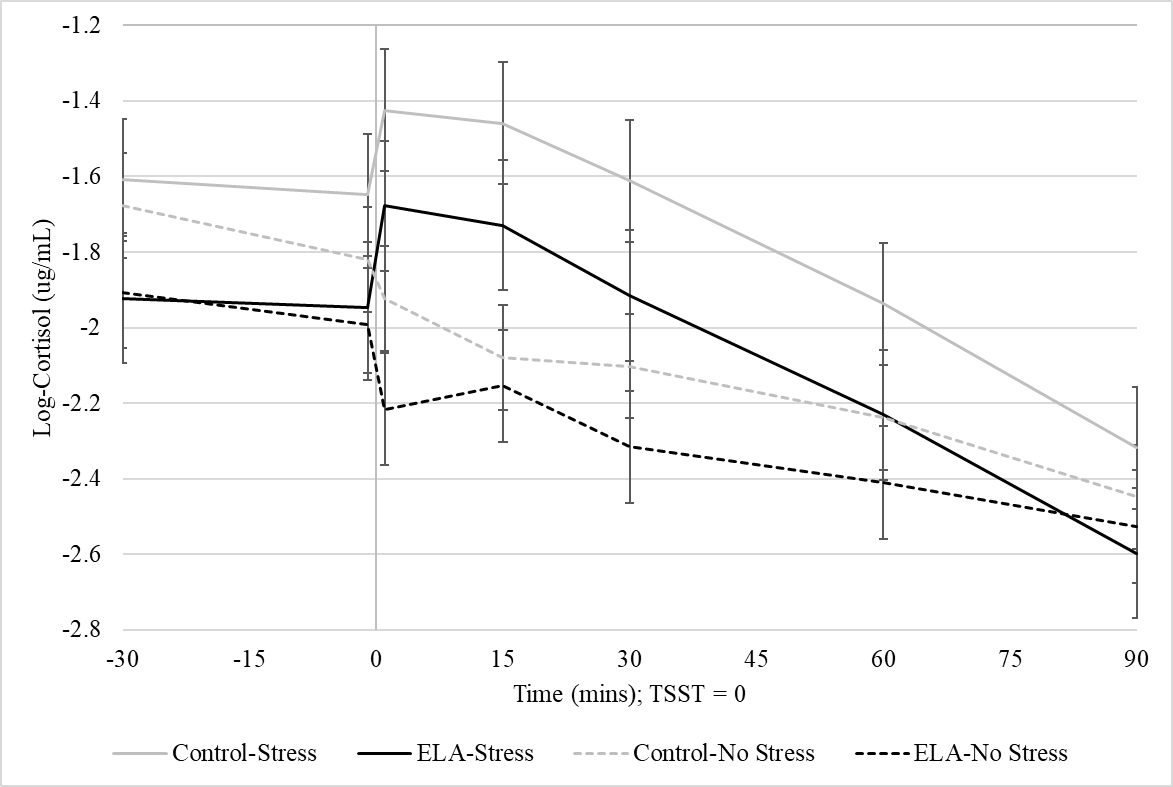


**Figure S1.** Log-Cortisol (Est±SE) across time within the ELA group vs. control group in the TSST and no-stress sessions. Time is denoted as minutes measured from the 15-minute long TSST or resting condition (0=midway point of session).

B. Mean Arterial Pressure (MAP) Analysis

*MAP Collection and Statistical Analysis*

Systolic and diastolic blood pressures were measured at 30 minutes after arrival, 1 minute prior to the experimental condition, 1 minute immediately after, and 15 minutes post-experimental condition. To understand the fast sympathetic response to the TSST, mean arterial pressure (MAP) was derived as a measure of average blood pressure response using the following equation:

$$MAP=\frac{\left[ \left( 2 X Pressure_{Diastolic} \right)+Pressure_{Systolic} \right]}{3}$$

MAP responses across time and session were modeled using repeated measures ANOVA in a multilevel modeling framework as described in the methods. MAP data was available for 34 participants (16 ELA, 18 non-ELA; total number of observations = 268; number of TSST session observations = 136). Tests for outliers did not identify any values greater than 3SD of mean values.

*Results:*

There was a significant within-person response to the TSST compared to the no-stress condition (Session, F=21.54, p<.0001; see Table S2), a significant change in MAP across time (Time, F=15.51, p<.0001), and a significant interaction between session and time (Session X Time, F=26.07, p<.0001). Exposure to the TSST was significantly associated with higher MAP at one minute prior to TSST exposure (b=7.46±2.22 *t*(93)=3.36, p=.001) and 1 minute post-TSST (b=7.94±2.22; *t*(93)=3.58, p=.0005). There was also a significant session by time interaction with ELA (Session X Time X ELA, F=3.21, p=.02). Differences by ELA-status manifested as greater increases in MAP in response to the TSST at one minute prior (b=7.99±3.26; *t*(93)=2.45, p=.01) and one minute post-TSST (b=8.88±3.26; *t*(93)=2.72, p=.007), as well as a continued elevation of MAP post-TSST at 15 minutes (b=7.79±3.26; *t*(93)=2.39, p=.01) compared to the control group.

The final simplified composite MAP model was of the form

| ${MAP}_{ij}=\beta_{0j}+\beta_{1}\left( {Session}_{j} \right)+\beta_{2}\left( {Time}_{ij} \right)+\beta_{3}\left( {Session}_{j} \right)\left( {Time}_{ij} \right)+\beta_{4}\left( {ELA}_{j} \right){+\beta_{5}\left( {Session}_{j} \right)\left( {ELA}_{j} \right) +{\beta_{6}\left( {Time}_{ij} \right)\left( {ELA}_{j} \right)+\beta}_{7}\left( {Time}_{ij} \right)\left( {Session}_{j} \right)\left( {ELA}_{j} \right)+\beta_{8}\left( {Age}_{j} \right)+\beta_{9}\left( {Sex}_{j} \right)+\beta_{10}\left( {Minority}_{j} \right)+\beta_{11}\left( {SES}_{j} \right)+\beta_{12}\left( {BMI}_{j} \right)+\mu_{0j}+\mu_{1j}\left( {Session}_{j} \right)+e}_{ti}$ | (1) |
| --- | --- |

| Table S2. Mean arterial pressure predicted by session and time (†p<.10, *p<.05, **p<.01, ***p<.001) | | | | | | | |
| --- | --- | --- | --- | --- | --- | --- | --- |
| **Mean arterial pressure (MAP)** | Model 1 | Model 2 | Model 3 | Model 4 | Model 5 | Model 6 | Final Model |
|  | Estimate (SE) | Estimate (SE) | Estimate (SE) | Estimate (SE) | Estimate (SE) | Estimate (SE) | Estimate (SE) |
| Fixed effects |  |  |  |  |  |  |  |
| **Intercept** | **89.83***(1.11)** | **84.89***(1.37)** | **88.39***(1.47)** | **87.52***(1.79)** | **87.19***(1.98)** | **87.19***(1.85)** | **86.03***(1.84)** |
| Session | -- | **5.82***(0.81)** | -1.06(1.47) | -1.07(1.47) | 1.06(1.96) | 1.06(2.22) | 1.06(2.22) |
| Time (-1 mins) | -- | **2.68*(1.14)** | **-3.00*(1.48)** | **-3.00*(1.48)** | -1.91(1.96) | -1.91(1.57) | -1.91(1.57) |
| Time (1 mins) | -- | **4.89***(1.14)** | -1.25(1.48) | -1.25(1.48) | -0.50(1.96) | -0.50(1.57) | -0.50(1.57) |
| Time (15 mins) | -- | 0.29(1.14) | -1.85(1.48) | -1.85(1.48) | -0.83(1.96) | -0.83(1.57) | -0.83(1.57) |
| Interaction: Session X Time (-1 mins) | -- | -- | **11.19***(2.08)** | **11.19***(2.08)** | **7.46**(2.78)** | **7.46**(2.22)** | **7.46**(2.22)** |
| Interaction: Session X Time (1 mins) | -- | -- | **12.10***(2.08)** | **12.10***(2.08)** | **7.94**(2.78)** | **7.94**(2.22)** | **7.94**(2.22)** |
| Interaction: Session X Time (15 mins) | -- | -- | **4.22*(2.08)** | **4.22*(2.08)** | 0.59(2.78) | 0.59(2.22) | 0.59(2.22) |
| ELA-status | -- | -- | -- | 1.84(2.18) | 2.57(2.91) | 2.41(2.73) | 3.88(2.91) |
| Interaction: Session X ELA | -- | -- | -- | -- | -4.52(2.89) | -4.36(2.86) | -4.31(3.26) |
| Interaction: ELA x Time (-1 mins) | -- | -- | -- | -- | -2.40(2.91) | -2.40(2.33) | -2.40(2.33) |
| Interaction: ELA x Time (1 mins) | -- | -- | -- | -- | -1.66(2.91) | -1.66(2.33) | -1.66(2.33) |
| Interaction: ELA x Time (15 mins) | -- | -- | -- | -- | -2.23(2.91) | -2.23(2.33) | -2.23(2.33) |
| Interaction: Session X ELA X Time (-1 mins) | -- | -- | -- | -- | **7.99†(4.08)** | **7.99*(3.26)** | **7.99*(3.26)** |
| Interaction: Session X ELA X Time (1 mins) | -- | -- | -- | -- | **8.88*(4.08)** | **8.88**(3.26)** | **8.88**(3.26)** |
| Interaction: Session X ELA X Time (15 mins) | -- | -- | -- | -- | **7.79†(4.08)** | **7.79*(3.26)** | **7.79*(3.26)** |
| Age | -- | -- | -- | -- | -- | -- | 0.72(.71) |
| Sex | -- | -- | -- | -- | -- | -- | 2.25(2.06) |
| Minority | -- | -- | -- | -- | -- | -- | 2.58(2.21) |
| SES | -- | -- | -- | -- | -- | -- | **3.60*(1.39)** |
| BMI | -- | -- | -- | -- | -- | -- | 0.16(.31) |
| Random effects |  |  |  |  |  |  |  |
| Subject-level Variance | 34.90(10.31) | 35.53(9.98) | 36.46(9.98) | 35.65(9.78) | 35.69(9.74) | 26.17(11.18) | 20.26(8.55) |
| Session-Intercept Covariance | -- | -- | -- | -- | -- | -0.02(9.45) | 0.61(8.34) |
| Session | -- | -- | -- | -- | -- | 30.65(13.78) | 35.13(13.70) |
| Residual Variance | 57.17(5.29) | 43.26(4.00) | 36.09(3.34) | 36.09(3.34) | 34.71(3.21) | 22.17(2.21) | 22.17(2.21) |
| AIC/BIC | 1910.6/1915.2 | 1852.4/1863.1 | 1816.1/1831.3 | 1817.4/1834.1 | 1822.1/1849.6 | 1779.2/1814.3 | 1781.8/1824.6 |
| LRT p-value | -- | -- | -- | -- | -- | p<.0001 | -- |

**
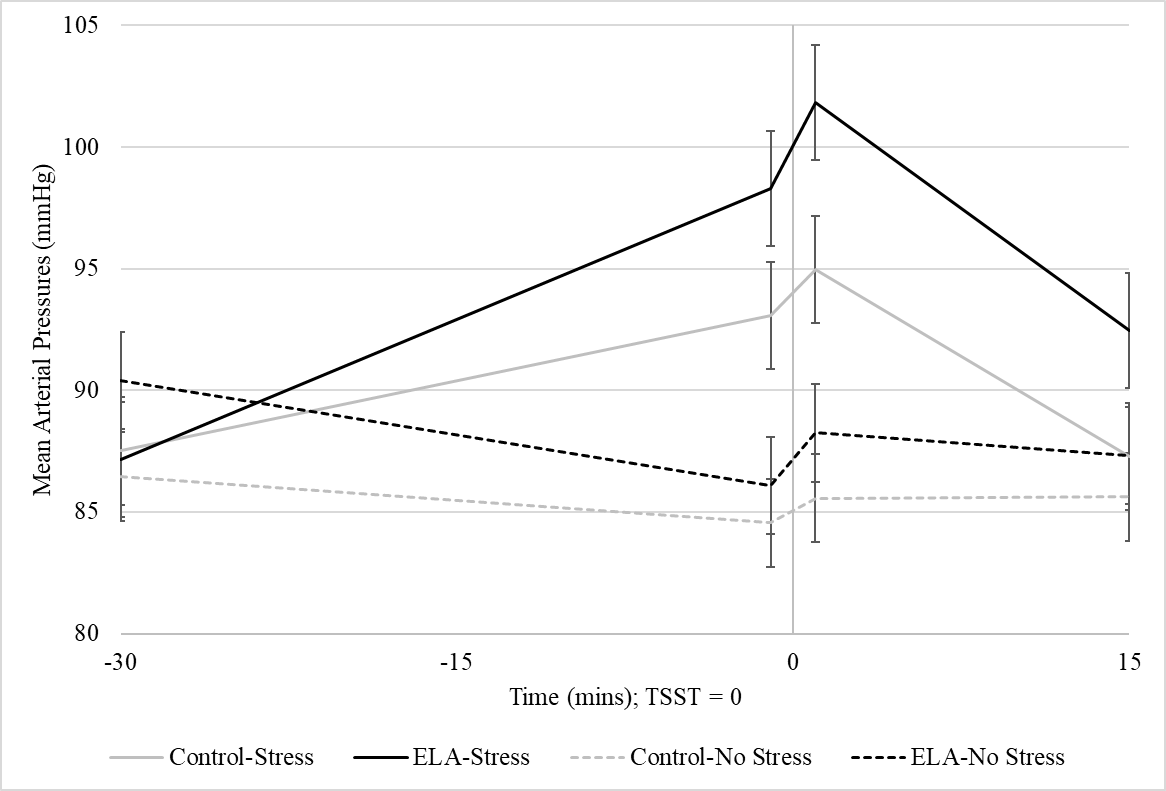
**

**Figure S2.** MAP (Est±SE) across time within the ELA group vs. control group in the TSST and no-stress sessions. Time is denoted as minutes measured from the 15-minute long TSST or resting condition (0=midway point of session).

C. Supplemental Tables

Table S3. Correlations among study variables (person-mean cell counts across all 8 time points)

|  | Age | ELA-Status | Sex (M) | Minority | BMI | SES | Total WBCs | Lymphocytes | Monocytes | Neutrophils | Eosinophils |
| --- | --- | --- | --- | --- | --- | --- | --- | --- | --- | --- | --- |
| ELA-Status | 0.18 |  |  |  |  |  |  |  |  |  |  |
| Sex(M) | -0.18 | -0.06 |  |  |  |  |  |  |  |  |  |
| Minority | -0.21 | -0.27 | -0.02 |  |  |  |  |  |  |  |  |
| BMI | 0.04 | **-0.52**** | 0.19 | -0.07 |  |  |  |  |  |  |  |
| SES | **-0.40*** | -0.09 | -0.17 | 0.17 | -0.09 |  |  |  |  |  |  |
| Total WBC | 0.22 | **0.34†** | **-0.35†** | -0.07 | -0.17 | -0.01 |  |  |  |  |  |
| Lymphocytes | 0.25 | 0.29 | -0.24 | -0.13 | **-0.34†** | 0.10 | **0.34†** |  |  |  |  |
| Monocytes | 0.21 | 0.13 | -0.20 | -0.16 | -0.08 | -0.13 | **0.66***** | 0.18 |  |  |  |
| Neutrophils | 0.10 | 0.27 | **-0.32†** | -0.03 | -0.10 | 0.01 | **0.95***** | 0.05 | **0.56**** |  |  |
| Eosinophils | 0.20 | -0.02 | **0.35†** | 0.16 | 0.18 | **-0.41*** | -0.06 | 0.16 | 0.09 | -0.21 |  |
| Basophils | **0.41*** | **0.49**** | **-0.53**** | -0.10 | **-0.40*** | -0.09 | **0.45*** | 0.25 | **0.42*** | **0.38*** | -0.26 |
| Notes: † p<.10, * p<.05, ** p<.01, *** p<.001. | | | | | | | | | | | |

Table S4. Immune cell counts means and standard deviations for sample across session and time

| Time | -30 mins | | | 30 mins | | | 90 mins | | | 240 mins | | |
| --- | --- | --- | --- | --- | --- | --- | --- | --- | --- | --- | --- | --- |
| Variable, M(SD) | Full Sample | Control-group | ELA-group | Full Sample | Control-group | ELA-group | Full Sample | Control-group | ELA-group | Full Sample | Control-group | ELA-group |
| TSST Session | | | | | | | | | | | | |
| WBC | 5.57 (1.4) | 5.25 (1.0) | 5.94 (1.7) | 6.06 (1.7) | 5.50 (1.1) | 6.70 (2.0) | 6.51 (1.8) | 5.96 (1.4) | 7.15 (1.9) | 6.66 (1.6) | 6.32 (1.2) | 7.03 (1.8) |
| Lymphocyte Count | 1663 (337) | 1574 (244) | 1766 (406) | 1620 (355) | 1519 (307) | 1737 (381) | 1660 (398) | 1552 (278) | 1777 (480) | 1999 (405) | 1847 (214) | 2151 (497) |
| Neutrophil Count | 3278 (1244) | 3097 (1022) | 3486 (1474) | 3831 (1509) | 3414 (1040) | 4312 (1842) | 4285 (1607) | 3914 (1376) | 4694 (1792) | 4082 (1342) | 3962 (1160) | 4202 (1545) |
| Monocyte Count | 487 (135) | 457 (106) | 522 (159) | 463 (157) | 447 (132) | 483 (185) | 468 (168) | 434 (131) | 505 (199) | 483 (149) | 488 (128) | 478 (172) |
| Eosinophil Count | 113 (87) | 105 (99) | 123 (74) | 109 (85) | 106 (104) | 113 (59) | 117 (118) | 101 (91) | 134 (143) | 125 (128) | 97 (103) | 153 (149) |
| Basophil Count | 28 (15) | 21 (12) | 35 (16) | 31 (20) | 23 (16) | 40 (20) | 34 (23) | 25 (16) | 44 (26) | 37 (21) | 34 (20) | 40 (23) |
| No-Stress Session | | | | | | | | | | | | |
| WBC | 5.35 (1.27) | 5.14 (1.2) | 5.58 (1.3) | 5.84 (1.6) | 5.34 (1.0) | 6.42 (1.9) | 6.12 (1.4) | 5.74 (0.9) | 6.56 (1.7) | 6.06 (1.3) | 5.67 (0.8) | 6.54 (1.6) |
| Lymphocyte Count | 1631 (354) | 1578 (336) | 1692 (378) | 1690 (397) | 1546 (338) | 1855 (406) | 1818 (343) | 1738 (371) | 1910 (295) | 2015 (638) | 1960 (779) | 2082 (427) |
| Neutrophil Count | 3076 (1150) | 2941 (1203) | 3231 (1112) | 3501 (1447) | 3162 (1121) | 3892 (1714) | 3629 (1383) | 3342 (1031) | 3961 (1685) | 3392 (1211) | 3035 (822) | 3831 (1483) |
| Monocyte Count | 467 (139) | 452 (116) | 484 (164) | 475 (141) | 461 (127) | 492 (159) | 489 (139) | 473 (139) | 509 (141) | 472 (142) | 490 (151) | 449 (133) |
| Eosinophil Count | 147 (106) | 143 (124) | 151 (87) | 145 (114) | 149 (140) | 142 (80) | 151 (126) | 162 (155) | 139 (85) | 148 (131) | 157 (162) | 136 (83) |
| Basophil Count | 28 (16) | 20 (9) | 37 (18) | 30 (19) | 21 (13) | 39 (22) | 32 (16) | 25 (13) | 39 (16 | 28 (16) | 21 (16) | 36 (13) |

Table S5. Model iterations for total white blood cell counts

| **Total white blood cell counts** | Model 1 | Model 2 | Model 3 | Model 4 | Model 5 | Model 6 | Final Model |
| --- | --- | --- | --- | --- | --- | --- | --- |
|  | Estimate (SE) | Estimate (SE) | Estimate (SE) | Estimate (SE) | Estimate (SE) | Estimate (SE) | Estimate (SE) |
| Fixed effects |  |  |  |  |  |  |  |
| Intercept | **5.99***(.24)** | **5.30***(.26)** | **5.49***(.24)** | **5.49***(.24)** | **5.48***(.24)** | **5.48***(.24)** | **5.41***(.28)** |
| Session | -- | **0.28**(.10)** | **0.28**(.10)** | 0.15(.19) | 0.16(.19) | 0.15(.22) | 0.14(.22) |
| Time (30 mins) | -- | **0.49**(.13)** | **0.49**(.13)** | **0.49**(.19)** | **0.51**(.13)** | **0.51***(.09)** | **0.51***(.09)** |
| Time (90 mins) | -- | **0.86***(.13)** | **0.86***(.13)** | **0.86***(.13)** | **0.87***(.13)** | **0.87***(.09)** | **0.87***(.09)** |
| Time (240 mins) | -- | **0.87***(.14)** | **0.87***(.14)** | **0.88**(.14)** | **0.88***(.13)** | **0.90***(.09)** | **0.90***(.10)** |
| Interaction: Session X Time (30 mins) | -- | -- | -- | -0.01(.27) | -0.01(.26) | -0.01(.19) | -0.01(.19) |
| Interaction: Session X Time (90 mins) | -- | -- | -- | 0.16(.27) | 0.17(.26) | 0.17(.19) | 0.17(.19) |
| Interaction: Session X Time (240 mins) | -- | -- | -- | 0.37(.27) | 0.35(.26) | **0.43*(.19)** | **0.43†(.19)** |
| ELA-status | -- | -- | **0.90†(.46)** | **0.90†(.46)** | 0.57(.48) | 0.55(.48) | 0.95(.69) |
| Interaction: Session X ELA | -- | -- | -- | -- | 0.39(.37) | 0.42(.44) | 0.42(.44) |
| Interaction: ELA x Time (30 mins) | -- | -- | -- | -- | **0.57*(.26)** | **0.57**(.19)** | **0.57**(.19)** |
| Interaction: ELA x Time (90 mins) | -- | -- | -- | -- | **0.44†(.26)** | **0.44*(.19)** | **0.44*(.19)** |
| Interaction: ELA x Time (240 mins) | -- | -- | -- | -- | 0.29(.27) | **0.35†(.19)** | **0.35†(.19)** |
| Interaction: Session X ELA X Time (30 mins) | -- | -- | -- | -- | -0.12(.53) | -0.12(.38) | -0.12(.38) |
| Interaction: Session X ELA X Time (90 mins) | -- | -- | -- | -- | 0.12(.53) | 0.12(.38) | 0.12(.38) |
| Interaction: Session X ELA X Time (240 mins) | -- | -- | -- | -- | -0.41(.53) | -0.36(.39) | -0.36(.39) |
| Age | -- | -- | -- | -- | -- | -- | -0.02(.19) |
| Sex | -- | -- | -- | -- | -- | -- | -0.97(.51) |
| Minority | -- | -- | -- | -- | -- | -- | 0.20(.49) |
| SES | -- | -- | -- | -- | -- | -- | 0.02(.31) |
| BMI | -- | -- | -- | -- | -- | -- | 0.09(.09) |
| Random effects |  |  |  |  |  |  |  |
| Subject-level Variance | 1.65(.46) | 1.64(.45) | 1.44(.40) | 1.45(.40) | 1.46(.40) | 1.51(.41) | 1.29(.35) |
| Session-Intercept Covariance | -- | -- | -- | -- | -- | 0.22(.23) | 0.22(.23) |
| Session | -- | -- | -- | -- | -- | 0.83(.26) | 0.83(.26) |
| Residual Variance | 0.67(.07) | 0.51(.05) | 0.51(.05) | 0.50(.05) | 0.48(.05) | 0.25(.03) | 0.25(.03) |
| AIC/BIC | 634/638 | 588/599 | 586/599 | 590/607 | 595/623 | 529/559 | 534/572 |
| LRT p-value | -- | -- | -- | -- | -- | p<.0001 | -- |

Table S6. Model iterations for lymphocyte counts

| **Lymphocyte counts** | Model 1 | Model 2 | Model 3 | Model 4 | Model 5 | Model 6 | Model 7 | Final Model |
| --- | --- | --- | --- | --- | --- | --- | --- | --- |
|  | Estimate (SE) | Estimate (SE) | Estimate (SE) | Estimate (SE) | Estimate (SE) | Estimate (SE) | Estimate (SE) | Estimate (SE) |
| Fixed effects |  |  |  |  |  |  |  |  |
| Intercept (Peak) | **1761*****  **(61)** | **1651*****  **(68)** | **1661*****  **(66)** | **1661*****  **(66)** | **1659*****  **(66)** | **1690*****  **(73)** | **1659*****  **(84)** | **1669*****  **(84)** |
| Session | -- | **-72*(36)** | **-72*(36)** | 11(70) | 14(69) | -44(101) | -48(101) | -57(101) |
| Time (30 mins) | -- | 8(50) | 8(50) | 8(49) | 12(49) | 12(33) | 12(33) | 8(33) |
| Time (90 mins) | -- | **87†(50)** | **87†(50)** | **86†(49)** | **87†(49)** | **87**(33)** | **87**(33)** | **85*(33)** |
| Time (240 mins) | -- | **353***(51)** | **353***(51)** | **356***(50)** | **356***(50)** | **324***(33)** | **324***(33)** | **321***(34)** |
| Interaction: Session X Time (30 mins) | -- | -- | -- | -102(98) | -108(98) | **-108†(65)** | **-108†(65)** | -102(65) |
| Interaction: Session X Time (90 mins) | -- | -- | -- | **-203*(99)** | **-204*(98)** | **-205**(65)** | **-205**(65)** | **-205**(67)** |
| Interaction: Session X Time (240 mins) | -- | -- | -- | -26(100) | -27(99) | 31(67) | 31(67) | 35(68) |
| ELA-status | -- | -- | 192(117) | 192(117) | 141(132) | 78(147) | -117(190) | 35(165) |
| Interaction: Session X ELA | -- | -- | -- | -- | 119(138) | 236(202) | 244(202) | -- |
| Interaction: ELA x Time (30 mins) | -- | -- | -- | -- | 111(98) | **111†(65)** | **111†(65)** | -- |
| Interaction: ELA x Time (90 mins) | -- | -- | -- | -- | 55(98) | 56(65) | 57(65) | -- |
| Interaction: ELA x Time (240 mins) | -- | -- | -- | -- | 48(100) | **116†(67)** | **119†(67)** | -- |
| Interaction: Session X ELA X Time (30 mins) | -- | -- | -- | -- | -168(195) | -168(130) | -168(130) | -- |
| Interaction: Session X ELA X Time (90 mins) | -- | -- | -- | -- | -6(196) | -4(130) | -2(130) | -- |
| Interaction: Session X ELA X Time (240 mins) | -- | -- | -- | -- | 16(199) | -89(133) | -84(133) | -- |
| Age | -- | -- | -- | -- | -- | -- | 56(50) | 57(50) |
| Sex | -- | -- | -- | -- | -- | -- | -111(129) | -106(129) |
| Minority | -- | -- | -- | -- | -- | -- | 4(126) | 10(127) |
| SES | -- | -- | -- | -- | -- | -- | 48(81) | 44(81) |
| BMI | -- | -- | -- | -- | -- | -- | -33(23) | -33(23) |
| Random effects |  |  |  |  |  |  |  |  |
| Subject-level Variance | 94856  (28273) | 98314  (28292) | 89007  (25891) | 89728  (26019) | 90251  (26124) | 138728  (38091) | 122566  (34612) | 122851  (35045) |
| Session-Intercept Covariance | -- | -- | -- | -- | -- | -106683  (42093) | -110923  (41486) | -111358  (42040) |
| Session | -- | -- | -- | -- | -- | 229727  (69474) | 228688  (69051) | 229497  (69686) |
| Residual Variance | 94450  (9665) | 69423  (7104) | 69438  (7107) | 67613  (6921) | 66285  (6785) | 29488  (3310) | 29476  (3308) | 30762  (3454) |
| AIC/BIC | 3213/3217 | 3162/3173 | 3161/3173 | 3162/3179 | 3173/3200 | 3100/3130 | 3103/3141 | 3096/3123 |
| LRT p-value | -- | -- | -- | -- | -- | p<.0001 | -- | -- |

Table S7. Model iterations for monocyte counts

| **Monocyte counts** | Model 1 | Model 2 | Model 3 | Model 4 | Model 5 | Model 6 | Final Model |
| --- | --- | --- | --- | --- | --- | --- | --- |
|  | Estimate (SE) | Estimate (SE) | Estimate (SE) | Estimate (SE) | Estimate (SE) | Estimate (SE) | Estimate (SE) |
| Fixed effects |  |  |  |  |  |  |  |
| Intercept | **474***(23)** | **475***(24)** | **476***(24)** | **476***(24)** | **477***(24)** | **475***(24)** | **475***(24)** |
| Session | -- | -1(11) | -1(11) | 19(21) | 20(21) | 23(23) | 23(23) |
| Time (30 mins) | -- | -8(15) | -8(15) | -8(15) | -8(15) | -8(13) | -8(13) |
| Time (90 mins) | -- | 3(15) | 3(15) | 2(15) | 2(15) | 1(13) | 1(13) |
| Time (240 mins) | -- | 4(15) | 4(15) | 5(15) | 2(15) | 2(13) | 2(13) |
| Interaction: Session X Time (30 mins) | -- | -- | -- | -32(30) | -33(29) | -33(26) | -33(26) |
| Interaction: Session X Time (90 mins) | -- | -- | -- | -41(30) | -42(29) | **-43†(26)** | **-43†(26)** |
| Interaction: Session X Time (240 mins) | -- | -- | -- | -4(30) | -2(30) | -7(27) | -7(27) |
| ELA-status | -- | -- | 31(45) | 31(45) | 52(48) | 55(48) | 27(71) |
| Interaction: Session X ELA | -- | -- |  | -- | 37(41) | 31(45) | 30(45) |
| Interaction: ELA x Time (30 mins) | -- | -- |  | -- | -14(29) | -14(26) | -14(26) |
| Interaction: ELA x Time (90 mins) | -- | -- |  | -- | 4(29) | 6(26) | 6(26) |
| Interaction: ELA x Time (240 mins) | -- | -- |  | -- | **-74*(30)** | **-71**(26)** | **-71**(26)** |
| Interaction: Session X ELA X Time (30 mins) | -- | -- |  | -- | -29(58) | -29(51) | -29(52) |
| Interaction: Session X ELA X Time (90 mins) | -- | -- |  | -- | -2(59) | -2(51) | 1(52) |
| Interaction: Session X ELA X Time (240 mins) | -- | -- |  | -- | 0(60) | 19(53) | 19(53) |
| Age | -- | -- |  | -- | -- | -- | 13(20) |
| Sex | -- | -- |  | -- | -- | -- | -27(53) |
| Minority | -- | -- |  | -- | -- | -- | -24(51) |
| SES | -- | -- |  | -- | -- | -- | -23(32) |
| BMI | -- | -- |  | -- | -- | -- | -3(10) |
| Random effects |  |  |  |  |  |  |  |
| Subject-level Variance | 13898  (3869) | 13913(3873) | 13679(3810) | 13697(3811) | 13774(3820) | 14017(3848) | 12873(3553) |
| Session-Intercept Covariance | -- | -- | -- | -- | -- | 1141(1972) | 224(2155) |
| Session | -- | -- | -- | -- | -- | 4916(2146) | 4964(2162) |
| Residual Variance | 6433(658) | 6409(656) | 6409(656) | 6319(646) | 5956(609) | 4683(526) | 4678(525) |
| AIC/BIC | 2642/2647 | 2649/2660 | 2651/2663 | 2654/2671 | 2657/2685 | 2645/2676 | 2654/2692 |
| LRT p-value | -- | -- | -- | -- | -- | p=.0004 | -- |

Table S8. Model iterations for neutrophil counts

| **Neutrophil counts** | Model 1 | Model 2 | Model 3 | Model 4 | Model 5 | Model 6 | Model 7 | Final Model |
| --- | --- | --- | --- | --- | --- | --- | --- | --- |
|  | Estimate (SE) | Estimate (SE) | Estimate (SE) | Estimate (SE) | Estimate (SE) | Estimate (SE) | Estimate (SE) | Estimate (SE) |
| Fixed effects |  |  |  |  |  |  |  |  |
| Intercept | **3594*****  **(220)** | **3154*****  **(233)** | **3154*****  **(233)** | **3186*****  **(226)** | **3177*****  **(226)** | **3171*****  **(217)** | **3102*****  **(251)** | **3103*****  **(251)** |
| Session | -- | **402***(96)** | 143(186) | 143(186) | 149(184) | 177(220) | 174(220) | 171(221) |
| Time (30 mins) | -- | **489*****  **(133)** | **489*****  **(131)** | **489*****  **(131)** | **506****  **(129)** | **506*****  **(91)** | **506*****  **(91)** | **506*****  **(92)** |
| Time (90 mins) | -- | **790*****  **(133)** | **792*****  **(131)** | **792*****  **(131)** | **802*****  **(130)** | **785*****  **(92)** | **785*****  **(92)** | **785*****  **(93)** |
| Time (240 mins) | -- | **524*****  **(135)** | **534*****  **(134)** | **534*****  **(134)** | **547*****  **(132)** | **570***(94)** | **569***(94)** | **566***(94)** |
| Interaction: Session X Time (30 mins) | -- | -- | 128(262) | 128(262) | 130(259) | 130(183) | 130(183) | 128(183) |
| Interaction: Session X Time (90 mins) | -- | -- | **478†(264)** | **477†(264)** | **474†(260)** | **440*(184)** | **440*(184)** | **438*(185)** |
| Interaction: Session X Time (240 mins) | -- | -- | **449†(267)** | **447†(267)** | 434(264) | **439*(188)** | **439*(188)** | **446*(188)** |
| ELA-status | -- | -- | -- | 629(423) | 364(452) | 375(434) | 1016(624) | 998(619) |
| Interaction: Session X ELA | -- | -- | -- | -- | 214(367) | 157(439) | 163(439) | -- |
| Interaction: ELA x Time (30 mins) | -- | -- | -- | -- | **474†(259)** | **474*(183)** | **474*(183)** | **474*(184)** |
| Interaction: ELA x Time (90 mins) | -- | -- | -- | -- | 323(260) | **358†(184)** | **358†(184)** | **358†(185)** |
| Interaction: ELA x Time (240 mins) | -- | -- | -- | -- | 248(264) | 294(188) | 296(188) | **312†(188)** |
| Interaction: Session X ELA X Time (30 mins) | -- | -- | -- | -- | 68(518) | 68(366) | 68(366) | -- |
| Interaction: Session X ELA X Time (90 mins) | -- | -- | -- | -- | -12(520) | 56(368) | 56(368) | -- |
| Interaction: Session X ELA X Time (240 mins) | -- | -- | -- | -- | -581(528) | -410(375) | -410(375) | -- |
| Age | -- | -- | -- | -- | -- | -- | -140(179) | -139(179) |
| Sex | -- | -- | -- | -- | -- | -- | **-985*(462)** | **-980*(462)** |
| Minority | -- | -- | -- | -- | -- | -- | 331(441) | 337(441) |
| SES | -- | -- | -- | -- | -- | -- | 24(282) | 22(283) |
| BMI | -- | -- | -- | -- | -- | -- | 127(84) | 127(84) |
| Random effects |  |  |  |  |  |  |  |  |
| Subject-level Variance | 1319748  (369138) | 1315622  (363025) | 1317267  (363037) | 1220068  (337283) | 1222771  (337581) | 1225944  (332358) | 1047278  (286445) | 1046217  (286159) |
| Session-Intercept Covariance | -- | -- | -- | -- | -- | 142758  (212908) | 212331  (207105) | 212794  (208364) |
| Session | -- | -- | -- | -- | -- | 874027  (269464) | 872336  (268881) | 884145  (272489) |
| Residual Variance | 629563  (64427) | 493070  (50455) | 481021  (49222) | 480979  (49213) | 467117  (47797) | 232990  (25814) | 233008  (25817) | 235672  (26115) |
| AIC/BIC | 3650/3654 | 3611/3621 | 3612/3627 | 3612/3629 | 3620/3648 | 3549/3579 | 3553/3592 | 3547/3579 |
| LRT p-value | -- | -- | -- | -- | -- | p<.0001 | -- | -- |

Table S9. Model iterations for log-eosinophil counts

| **Log-transformed eosinophil counts** | Model 1 | Model 2 | Model 3 | Model 4 | Model 5 | Model 6 | Model 7 | Final Model |
| --- | --- | --- | --- | --- | --- | --- | --- | --- |
|  | Estimate (SE) | Estimate (SE) | Estimate (SE) | Estimate (SE) | Estimate (SE) | Estimate (SE) | Estimate (SE) | Estimate (SE) |
| Fixed effects |  |  |  |  |  |  |  |  |
| Intercept | **4.58*****  **(.15)** | **4.60*****  **(.15)** | **4.60*****  **(.15)** | **4.61*****  **(.15)** | **4.61*****  **(.16)** | **4.61*****  **(.15)** | **4.97*****  **(.14)** | **4.97*****  **(.14)** |
| Session | **--** | **-0.21*****  **(.05)** | **-0.24*(.10)** | **-0.24*(.10)** | **-0.24*(.10)** | **-0.24*(.10)** | **-0.23*(.11)** | **-0.21*(.08)** |
| Time (30 mins) | -- | -0.02(.07) | -0.02(.07) | -0.02(.07) | -0.02(.07) | -0.02(.05) | -0.02(.05) | -0.02(.06) |
| Time (90 mins) | -- | -0.03(.07) | -0.03(.07) | -0.03(.07) | -0.03(.07) | -0.03(.05) | -0.04(.05) | -0.03(.06) |
| Time (240 mins) | -- | -0.04(.07) | -0.04(.07) | -0.03(.07) | -0.04(.07) | -0.05(.06) | -0.05(.06) | -0.05(.06) |
| Interaction: Session X Time (30 mins) | -- | -- | 0.02(.13) | 0.02(.14) | 0.02(.13) | 0.02(.11) | 0.02(.11) | -- |
| Interaction: Session X Time (90 mins) | -- | -- | -0.02(.13) | -0.02(.14) | -0.01(.13) | -0.02(.11) | -0.02(.11) | -- |
| Interaction: Session X Time (240 mins) | -- | -- | 0.14(14) | 0.14(.14) | 0.14(.14) | 0.10(.11) | 0.10(.11) | -- |
| ELA-status | -- | -- | -- | 0.21(.29) | 0.28(.30) | 0.30(.30) | 0.47(.35) | 0.39(.35) |
| Interaction: Session X ELA | -- | -- | -- | -- | 0.05(.19) | 0.04(.21) | 0.02(.21) | -- |
| Interaction: ELA x Time (30 mins) | -- | -- | -- | -- | -0.07(.13) | -0.07(.11) | -0.07(.11) | -- |
| Interaction: ELA x Time (90 mins) | -- | -- | -- | -- | -0.08(.13) | -0.08(.11) | -0.08(.11) | -- |
| Interaction: ELA x Time (240 mins) | -- | -- | -- | -- | -0.17(.14) | -0.17(.11) | -0.17(.11) | -- |
| Interaction: Session X ELA X Time (30 mins) | -- | -- | -- | -- | -0.04(.27) | -0.04(.22) | -0.04(.22) | -- |
| Interaction: Session X ELA X Time (90 mins) | -- | -- | -- | -- | 0.10(.27) | 0.10(.22) | 0.12(.22) | -- |
| Interaction: Session X ELA X Time (240 mins) | -- | -- | -- | -- | -0.01(.27) | -0.01(.22) | 0.02(.22) | -- |
| Age | -- | -- | -- | -- | -- | -- | **0.28*(.11)** | **0.28*(.10)** |
| Sex | -- | -- | -- | -- | -- | -- | **0.75****  **(.26)** | **0.75**(.26)** |
| Minority | -- | -- | -- | -- | -- | -- | 0.43(.25) | 0.43(.25) |
| SES | -- | -- | -- | -- | -- | -- | **-0.41*(.16)** | **-0.41*(.16)** |
| BMI | -- | -- | -- | -- | -- | -- | 0.02(.05) | 0.02(.05) |
| Random effects |  |  |  |  |  |  |  |  |
| Subject-level Variance | 0.61(.17) | 0.60(.16) | 0.60(.16) | 0.59(.16) | 0.63(.18) | 0.59(.16) | 0.34(.09) | 0.34(.09) |
| Session-Intercept Covariance | -- | -- | -- | -- | -- | 0.03(.07) | 0.04(.05) | 0.04(.05) |
| Session | -- | -- | -- | -- | -- | 0.15(.05) | 0.15(.05) | 0.15(.05) |
| Residual Variance | 0.14(.01) | 0.13(.01) | 0.13(.01) | 0.13(.01) | 0.12(.01) | 0.08(.009) | 0.08(.009) | 0.08(.009) |
| AIC/BIC | 299/303 | 289/300 | 293/309 | 295/312 | 307/334 | 274/305 | 268/306 | 252/275 |
| LRT p-value | -- | -- | -- | -- | -- | p<.0001 | -- | -- |

Table S10. Model iterations for basophil counts

| **Basophil counts** | Model 1 | Model 2 | Model 3 | Model 4 | Model 5 | Model 6 | Model 7 | Final Model |
| --- | --- | --- | --- | --- | --- | --- | --- | --- |
|  | Estimate (SE) | Estimate (SE) | Estimate (SE) | Estimate (SE) | Estimate (SE) | Estimate (SE) | Estimate (SE) | Estimate (SE) |
| Fixed effects |  |  |  |  |  |  |  |  |
| Intercept | **29.9*****  **(3.0)** | **27.1*****  **(3.2)** | **27.1*****  **(3.2)** | **27.9*****  **(2.9)** | **27.9*****  **(2.8)** | **27.9*****  **(2.8)** | **28.6*****  **(2.8)** | **28.6*****  **(2.8)** |
| Session | -- | **2.6*(1.1)** | 0.2(2.3) | 0.2(2.3) | 0.1(2.2) | -0.1(2.3) | -0.2(2.3) | -0.1(2.3) |
| Time (30 mins) | -- | 2.6(1.6) | 2.6(1.6) | 2.6(1.6) | **2.7†(1.6)** | **2.7†(1.5)** | **2.7†(1.5)** | **2.7†(1.5)** |
| Time (90 mins) | -- | **5.1**(1.6)** | **5.1**(1.6)** | **5.1**(1.6)** | **5.1**(1.6)** | **5.0**(1.5)** | **5.0**(1.5)** | **5.0**(1.5)** |
| Time (240 mins) | -- | **4.1*(1.7)** | **4.4**(1.7)** | **4.3**(1.6)** | **4.3**(1.6)** | **4.3**(1.5)** | **4.3**(1.5)** | **4.3**(1.5)** |
| Interaction: Session X Time (30 mins) | -- | -- | 1.3(3.2) | 1.3(3.2) | 1.4(3.1) | 1.4(3.0) | 1.4(3.0) | 1.3(3.1) |
| Interaction: Session X Time (90 mins) | -- | -- | 1.5(3.2) | 1.5(3.2) | 1.8(3.1) | 1.7(3.0) | 1.8(3.0) | 1.5(3.1) |
| Interaction: Session X Time (240 mins) | -- | -- | **7.3*(3.3)** | **7.3*(3.3)** | **7.5*(3.2)** | **7.4*(3.0)** | **7.4*(3.1)** | **7.3*(3.1)** |
| ELA-status | -- | -- | -- | **16.0****  **(5.4)** | **16.3****  **(5.7)** | **16.3****  **(5.7)** | 9.5(6.7) | 9.9(6.0) |
| Interaction: Session X ELA | -- | -- | -- | -- | -3.9(4.4) | -3.7(4.6) | -3.5(4.6) | -- |
| Interaction: ELA x Time (30 mins) | -- | -- | -- | -- | 1.8(3.1) | 1.8(3.0) | 1.8(3.0) | -- |
| Interaction: ELA x Time (90 mins) | -- | -- | -- | -- | 1.5(3.1) | 1.6(3.0) | 1.6(3.0) | -- |
| Interaction: ELA x Time (240 mins) | -- | -- | -- | -- | -5.1(3.2) | -4.8(3.0) | -4.8(3.0) | -- |
| Interaction: Session X ELA X Time (30 mins) | -- | -- | -- | -- | 3.2(6.3) | 3.2(6.0) | 3.2(6.0) | -- |
| Interaction: Session X ELA X Time (90 mins) | -- | -- | -- | -- | 9.9(6.2) | **10.0†(6.0)** | **10.0†(6.0)** | -- |
| Interaction: Session X ELA X Time (240 mins) | -- | -- | -- | -- | -3.6(6.4) | -3.2(6.1) | -3.4(6.1) | -- |
| Age | -- | -- | -- | -- | -- | -- | 2.9(1.8) | 2.8(1.8) |
| Sex | -- | -- | -- | -- | -- | -- | **-10.7*(4.6)** | **-10.6*(4.6)** |
| Minority | -- | -- | -- | -- | -- | -- | 1.2(4.4) | 1.1(4.4) |
| SES | -- | -- | -- | -- | -- | -- | -4.5(2.8) | -4.6(2.8) |
| BMI | -- | -- | -- | -- | -- | -- | -0.9(.84) | -0.9(.84) |
| Random effects |  |  |  |  |  |  |  |  |
| Subject-level Variance | 262(72) | 261(71) | 260(71) | 197(54) | 198(54) | 199(54) | 129(37) | 129(37) |
| Session-Intercept Covariance | -- | -- | -- | -- | -- | 44(21) | 43(19) | 44(19) |
| Session | -- | -- | -- | -- | -- | 21(14) | 21(15) | 20(15) |
| Residual Variance | 80(8) | 74(8) | 72(7) | 72(7) | 68(7) | 62(7) | 62(7) | 66(7) |
| AIC/BIC | 1688/1693 | 1681/1692 | 1682/1697 | 1676/1693 | 1679/1706 | 1675/1705 | 1670/1708 | 1667/1694 |
| LRT p-value | -- | -- | -- | -- | -- | p=0.01 | -- | -- |

Table S11. The final simplified composite model for each cell type is as follows:

| ${WBC}_{ti}=\beta_{0j}+\beta_{1}\left( {Session}_{j} \right)+\beta_{2}\left( {Time}_{ij} \right)+\beta_{3}\left( {Session}_{j} \right)\left( {Time}_{ij} \right)+\beta_{4}\left( {ELA}_{j} \right){+\beta_{5}\left( {Session}_{j} \right)\left( {ELA}_{j} \right)+{\beta_{6}\left( {Time}_{ij} \right)\left( {ELA}_{j} \right)+\beta}_{7}\left( {Time}_{ij} \right)\left( {Session}_{j} \right)\left( {ELA}_{j} \right)+\beta_{8}\left( {Age}_{j} \right)+\beta_{9}\left( {Sex}_{j} \right)+\beta_{10}\left( {Minority}_{j} \right)+\beta_{11}\left( {SES}_{j} \right)+\beta_{12}\left( {BMI}_{j} \right)+\mu_{0j}+\mu_{1j}\left( {Session}_{j} \right)+e}_{ti}$ | (1) |
| --- | --- |
| ${Lymphocytes}_{ti}=\beta_{0j}+\beta_{1}\left( {Session}_{j} \right)+\beta_{2}\left( {Time}_{ij} \right)+\beta_{3}\left( {Session}_{j} \right)\left( {Time}_{ij} \right)+\beta_{4}\left( {ELA}_{j} \right){+\beta_{5}\left( {Age}_{j} \right)+\beta_{6}\left( {Sex}_{j} \right)+\beta_{7}\left( {Minority}_{j} \right)+\beta_{8}\left( {SES}_{j} \right)+\beta_{9}\left( {BMI}_{j} \right)+\mu_{0j}+\mu_{1j}\left( {Session}_{j} \right)+e}_{ti}$ | (2) |
| ${Monocytes}_{ti}=\beta_{0j}+\beta_{1}\left( {Session}_{j} \right)+\beta_{2}\left( {Time}_{ij} \right)+\beta_{3}\left( {Session}_{j} \right)\left( {Time}_{ij} \right)+\beta_{4}\left( {ELA}_{j} \right){+\beta_{5}\left( {Session}_{j} \right)\left( {ELA}_{j} \right)+{\beta_{6}\left( {Time}_{ij} \right)\left( {ELA}_{j} \right)+\beta}_{7}\left( {Time}_{ij} \right)\left( {Session}_{j} \right)\left( {ELA}_{j} \right)+\beta_{8}\left( {Age}_{j} \right)+\beta_{9}\left( {Sex}_{j} \right)+\beta_{10}\left( {Minority}_{j} \right)+\beta_{11}\left( {SES}_{j} \right)+\beta_{12}\left( {BMI}_{j} \right)+\mu_{0j}+\mu_{1j}\left( {Session}_{j} \right)+e}_{ti}$ | (3) |
| ${Neutrophils}_{ti}=\beta_{0j}+\beta_{1}\left( {Session}_{j} \right)+\beta_{2}\left( {Time}_{ij} \right)+\beta_{3}\left( {Session}_{j} \right)\left( {Time}_{ij} \right)+\beta_{4}\left( {ELA}_{j} \right){+\beta_{5}\left( {Time}_{ij} \right)\left( {ELA}_{j} \right)+\beta_{6}\left( {Age}_{j} \right)+\beta_{7}\left( {Sex}_{j} \right)+\beta_{8}\left( {Minority}_{j} \right)+\beta_{9}\left( {SES}_{j} \right)+\beta_{10}\left( {BMI}_{j} \right)+\mu_{0j}+\mu_{1j}\left( {Session}_{j} \right)+e}_{ti}$ | (4) |
| ${Log\text{-}Eosinophils}_{ti}=\beta_{0j}+\beta_{1}\left( {Session}_{j} \right)+\beta_{2}\left( {Time}_{ij} \right)+\beta_{3}\left( {Session}_{j} \right)\left( {Time}_{ij} \right)+\beta_{4}\left( {ELA}_{j} \right){+\beta_{5}\left( {Session}_{j} \right)\left( {ELA}_{j} \right)+{\beta_{6}\left( {Time}_{ij} \right)\left( {ELA}_{j} \right)+\beta}_{7}\left( {Time}_{ij} \right)\left( {Session}_{j} \right)\left( {ELA}_{j} \right)+\beta_{8}\left( {Age}_{j} \right)+\beta_{9}\left( {Sex}_{j} \right)+\beta_{10}\left( {Minority}_{j} \right)+\beta_{11}\left( {SES}_{j} \right)+\beta_{12}\left( {BMI}_{j} \right)+\mu_{0j}+\mu_{1j}\left( {Session}_{j} \right)+e}_{ti}$ | (5) |
| ${Basophils}_{ti}=\beta_{0j}+\beta_{1}\left( {Session}_{j} \right)+\beta_{2}\left( {Time}_{ij} \right)+\beta_{3}\left( {Session}_{j} \right)\left( {Time}_{ij} \right)+\beta_{4}\left( {ELA}_{j} \right){+\beta_{5}\left( {Age}_{j} \right)+\beta_{6}\left( {Sex}_{j} \right)+\beta_{7}\left( {Minority}_{j} \right)+\beta_{8}\left( {SES}_{j} \right)+\beta_{9}\left( {BMI}_{j} \right)+\mu_{0j}+\mu_{1j}\left( {Session}_{j} \right)+e}_{ti}$ | (6) |

1. () Nestor L. Lopez-Duran, Stefanie E. Mayer, and James L. Abelson, “Modeling Neuroendocrine Stress Reactivity in Salivary Cortisol: Adjusting for Peak Latency Variability,” *Stress* 17, no. 4 (July 2014): 285–95, https://doi.org/10.3109/10253890.2014.915517. [↑](#footnote-ref-1)
